# Microbial communities in paddy soils: differences in abundance and functionality between rhizosphere and pore water, the influence of different soil organic carbon, sulfate fertilization and cultivation time, and contribution to arsenic mobility and speciation

**DOI:** 10.1093/femsec/fiad121

**Published:** 2023-10-06

**Authors:** Sarah Zecchin, Jiajia Wang, Maria Martin, Marco Romani, Britta Planer-Friedrich, Lucia Cavalca

**Affiliations:** Dipartimento di Scienze per gli Alimenti, la Nutrizione e l'Ambiente (DeFENS), Università degli Studi di Milano, Milano-20133, Italy; Environmental Geochemistry Group, Bayreuth Center for Ecology and Environmental Research (BAYCEER), Bayreuth University, 95440, Germany; Department of Agriculture, Forest and Food Science, University of Turin, Turin-10095, Italy; Rice Research Centre, Ente Nazionale Risi, Castello d'Agogna, Pavia-27030, Italy; Environmental Geochemistry Group, Bayreuth Center for Ecology and Environmental Research (BAYCEER), Bayreuth University, 95440, Germany; Dipartimento di Scienze per gli Alimenti, la Nutrizione e l'Ambiente (DeFENS), Università degli Studi di Milano, Milano-20133, Italy

**Keywords:** arsenic thiolation, pore water microbiome, rhizosphere microbiome, rice paddy soil, sulfate fertilization

## Abstract

Abiotic factors and rhizosphere microbial populations influence arsenic accumulation in rice grains. Although mineral and organic surfaces are keystones in element cycling, localization of specific microbial reactions in the root/soil/pore water system is still unclear. Here, we tested if original unplanted soil, rhizosphere soil and pore water represented distinct ecological microniches for arsenic-, sulfur- and iron-cycling microorganisms and compared the influence of relevant factors such as soil type, sulfate fertilization and cultivation time. In rice open-air-mesocosms with two paddy soils (2.0% and 4.7% organic carbon), Illumina 16S rRNA gene sequencing demonstrated minor effects of cultivation time and sulfate fertilization that decreased Archaea-driven microbial networks and incremented sulfate-reducing and sulfur-oxidizing bacteria. Different compartments, characterized by different bacterial and archaeal compositions, had the strongest effect, with higher microbial abundances, bacterial biodiversity and interconnections in the rhizosphere vs pore water. Within each compartment, a significant soil type effect was observed. Higher percentage contributions of rhizosphere dissimilatory arsenate- and iron-reducing, arsenite-oxidizing, and, surprisingly, dissimilatory sulfate-reducing bacteria, as well as pore water iron-oxidizing bacteria in the lower organic carbon soil, supported previous chemistry-based interpretations of a more active S-cycling, a higher percentage of thioarsenates and lower arsenic mobility by sorption to mixed Fe(II)Fe(III)-minerals in this soil.

## Teaser

Rice paddy pore water and rhizosphere soil host completely different microbial populations, which drive arsenic concentration and thiolation.

## Introduction

Arsenic accumulates more in rice than in other crops, posing health concerns at a global level (Meharg et al. 2009). In aerobic soil environments, most of the metalloid is immobilized as arsenate in Fe (oxyhydr)oxides, while in flooded rice soils it is released mainly as arsenite by reductive dissolution.

Microorganisms methylate inorganic arsenic species to the less toxic mono- (MMA) and dimethylated (DMA) oxyarsenates, which are also taken up by the plants from paddy soil porewater (Meharg and Zhao 2012). Recently, thiolated arsenic forms have also been detected, both in paddy soil pore water (Wang et al. 2020b) and in rice grains (Colina Blanco et al. 2021). The reactions controlling the extent of arsenic dissolution and conversion into different chemical species depend on soil geochemical and physical factors and are often microbially mediated. Particularly, the plant rhizosphere in the paddy fields is characterized by steep gradients of redox conditions and physicochemical characteristics (pH, organic matter content and redox-sensitive elements, such as arsenic, sulfur and iron) that shape microbial community, even at microscale level.

Water management of the rice paddy was shown to strongly affect arsenic biogeochemistry by favoring specific microbial populations, which can actively convert the different metalloid oxidation states. In rice field soil, continuous flooding promotes the presence of arsenic-solubilizing ferric iron- and arsenate-reducing bacteria (Zecchin et al. 2017a,b, 2019), while in aerobic rice field soil the predominance of ferrous iron- and arsenite-oxidizing bacteria leads to arsenic immobilization on the solid phase, lowering its concentrations in the pore water and in rice grains (Xu et al. 2008, Arao et al. 2009, Zecchin et al. 2017b, Li et al. 2019).

Besides water management, sulfate fertilization is a promising tool to decrease arsenic contamination in rice grain, acting both at the plant (i.e. synthesis of phytochelatins) and at the soil level (Dahlawi et al. 2018, Zou et al. 2018, Chen et al. 2019, Hu et al. 2007, 2021, Fang et al. 2023). The decreased concentration of arsenic in the pore water of sulfate-amended rice paddy soil was positively related to the presence of rhizospheric dissimilatory sulfate-reducing microorganisms (DSRM) (Jia et al. 2015) that, by producing sulfide in anoxic conditions at circumneutral pH, contribute to the removal of arsenic by secondary iron sulfides (Hu et al. 2007, Burton et al. 2014, Xu et al. 2019). Part of sulfide is used by sulfur-oxidizing bacteria (SOB), which contribute to the production of elemental sulfur (S^0^) in rice paddies (Stubner et al. 1998, Zhou et al. 2002, Friedrich et al. 2005, Hamilton et al. 2014). Moreover, sulfide and S^0^ are hypothesized to react abiotically with either arsenite or methylated arsenates, to yield different inorganic and methylated thioarsenates (Planer-Friedrich et al. 2015, Fan et al. 2018, Wang et al. 2020b).

Wang et al. (2020b) suggested that soil organic carbon (C) content plays an important role in the biogeochemistry of arsenic by fueling microbial activity. In their study, the authors observed that sulfate addition caused a stronger decrease of dissolved arsenic coupled to higher percentage of methylation and thiolation in a low C soil compared with a high C soil. The hypothesis was that in the high C soil, reducing conditions lead to FeS mineral formation, a relatively large removal of reduced sulfur from the pore water and less active sulfur-cycling. By contrast, the lower C content caused less pronounced reducing conditions [with higher Eh and less Fe(II) in the porewater], with consequently a higher conversion of sulfide to S^0^ and finally sulfate and increased adsorption of arsenic to mixed valence iron minerals. The oxidized sulfur would then be available again for new organic C driven reduction, promoting an active sulfur cycling.

The localization of arsenic, sulfur and iron biogeochemical reaction sites in the soil/root/pore water rice paddy system is an important but still overlooked aspect. In fact, it is not clear whether arsenic, sulfur and iron transformations occur in solution or in the solid phases and which types of microbial populations are crucial in regulating these reactions. Moreover, while the composition of the microbial communities inhabiting different soil/plant compartments (i.e. bulk soil, rhizosphere soil, rhizoplane, endosphere) have been revealed by several authors (Somenahally et al. 2011, Zecchin et al. 2017a,b, Jia et al. 2014, Das et al. 2016), to date, the microbial communities living in rice paddy pore water have never been characterized, and their composition and role in element cycling is still unknown. A previous study (Tian et al. 2021) suggested that in wetlands the water table level is positively related to microbial species richness and diversity in the pore water. In light of recent issues with water scarcity, which are driving the consideration of novel water-saving agronomic regimes, the ecological equilibrium of keystone arsenic-, sulfur- and iron-cycling microbial species in the pore water can be altered. In order to clarify if compartmentalization is a major driver of microbial communities involved in arsenic, sulfur and iron biogeochemistry in rice paddies, in the present study we characterized bacterial and archaeal populations inhabiting the original unplanted soil, the rhizosphere soil and the porewater of two rice paddy soils with different organic C content, non-fertilized and fertilized with sulfate, and tested whether the compartment effect leads to stronger differences in comparison with other factors such as sulfate fertilization, soil type (with low and high organic C) and cultivation time. Vice versa, the possible influence of the different microbial communities on the geochemical parameters was statistically evaluated to determine the role of specific microbial populations in arsenic, sulfur and iron cycling, focusing on total arsenic mobility and speciation, specifically thiolation and methylation.

## Materials and methods

### Experimental set-up

The rice growing experiment was carried out at the Rice Research Center (Ente Nazionale Risi, ENR) in Castello d'Agogna (Pavia, Italy). The mesocosms were set up in the open air in 0.83 m^2^ plastic tanks filled with 30 cm of soil from two distinct paddy fields located in Cascina Fornazzo and Cascina Veronica (Pavia, hereafter referred to as "Fornazzo" and "Veronica" soils, respectively). Fornazzo and Veronica soils were taken as representatives of high and low C soils, being characterized by 47 and 20 g kg^−1^ of organic C, respectively (Wang et al. 2020a). Arsenic concentrations were similar between the two soils with 5.6 and 5.8 mg kg^−1^, respectively, which is below the Italian national limit for public use soil (20 mg kg^−1^, D.Lgs. 152/2006). Furthermore, Fornazzo soil had slightly higher total S and Fe(II) contents in comparison with Veronica soil (see Supplementary Table 1 for the complete characterization of the two soils). Absolute concentrations of dissolved total S and Fe(II) were lower in Veronica than in Fornazzo pore water (Supplementary Table 2), which reflected on the one hand the differences in total S and Fe contents in the two soils (Supplementary Table 1). However, the proportion of Fe mobilized from soil to pore water was similar for both soils, while the proportion of S mobilized from Veronica soil was lower than from Fornazzo soil (Supplementary Table 3), reflecting a higher overall redox potential in Veronica soil, as described before (Wang et al. 2020b).

Rice plants (*Oryza sativa* var. Selenio) were water-seeded and cultivated under continuous flooding for the whole life cycle, using non-sterile tap water provided with a garden hose. Before seeding, mesocosms were fertilized with 100 kg ha^−1^ of either ammonium sulfate [(NH_4_)_2_SO_4_] or urea (CH_4_N_2_O) as control nitrogen fertilizer. Further fertilization was applied at the tillering stage with 30 kg ha^−1^ and at the booting stage with 50 kg ha^−1^ of either urea or ammonium sulfate, according to the usual agronomic practices. For each type of fertilization (i.e. ammonium sulfate vs urea/control) and for each soil (i.e. Fornazzo vs Veronica), three replicates were set up. The physicochemical analyses were performed in the pore water over time by Wang et al. (2020a) and the results are summarized in Supplementary Tables 2 and 4.

### Rhizosphere soil separation and DNA isolation

To analyze the microbial communities inhabiting the rice rhizospheric compartment, rhizosphere soil (i.e. soil strictly attached to the roots) and pore water were collected during stem elongation, flowering and the dough stage (corresponding to approximately 60, 80 and 100 days after seeding, respectively). These three rice life stages are considered crucial for both the development of rhizospheric microbial communities on expanding roots (Edwards et al. 2018) and for arsenic uptake, which is highest during flowering (Zheng et al. 2011). The original unplanted soil was sampled for the characterization of the starting microbial community. For each experimental replicate, three plants were collected and pooled in one composite sample. Roots were shaken in tetrasodium pyrophosphate and the rhizosphere soil was separated from roots according to Zecchin et al. (2017b). Pore water was sampled with 15 µm-pore size Rhizon samplers (Rhizon SMS 5 cm, Rhizosphere, Wageningen, The Netherlands) and planktonic cells were collected on cellulose acetate filters (0.2 µm pores) with a vacuum pump. DNA was isolated from all samples using DNeasy PowerSoil kit (QIAGEN, Hilden, Germany), according to the manufacturer's instructions. The quality of the isolated DNA was checked under UV light by agarose gel electrophoresis on a 1% Tris-acetate-EDTA (TAE) agarose gel stained with GelRed (Biotium, CA, USA).

### Illumina 16S rRNA genes libraries

From DNA isolated from the original soils, rhizosphere soil and pore water samples, bacterial and archaeal 16S rRNA genes were sequenced with primers 341F/806R (5′- CCTACGGGAGGCAGCAG-3′/5′- GGACTACHVGGGTWTCTAAT-3′) and 344F/806R (5′-CCCTAYGGGGYGCASCAG-3′), respectively (Rago et al. 2017). Sequencing was performed on 1 and 0.1 µg of DNA for rhizosphere soil and pore water, respectively, at the DNA Services (DNAS) facility, Research Resources Center (RRC), University of Illinois at Chicago (UIC, USA). Raw reads were processed and analyzed with QIIME2 (https://qiime2.org/, Bolyen et al. 2019). The DADA2 workflow (Callahan et al. 2016) was used to remove barcodes and sequence adapters, filter high quality non-chimeric reads, cluster the reads in single amplicon sequence variants (ASVs) and pick one representative sequence for each ASV. Alpha diversity was estimated upon rarefaction of the datasets. Microbial species richness was determined by calculating the number of observed microbial species and using the Chao1 richness estimator (Chao, 1984), while microbial species evenness was estimated according to Pielou's algorithm (Pielou 1966). The taxonomy of representative sequences was assigned using the SILVA SSU reference dataset version 138 (https://www.arb-silva.de/). The taxonomic classification was performed using a naïve Bayes classifier optimized for the primers used in the sequencing process (Pedregosa et al. 2011, Bokulich et al. 2018). ASV tables were obtained to determine the relative abundance of each taxon in the samples. Representative sequences were aligned with mafft (Katoh and Standley 2013) and phylogenetic analysis of the representative sequences was performed with FastTree (Price et al. 2010).

### Functional prediction

The presence in the library of microorganisms related to arsenic, sulfur and iron cycles and in methanogenesis [i.e. dissimilatory arsenate reducing bacteria (DAsRB), arsenate-reducing bacteria (AsRB), arsenite-oxidizing bacteria (AsOB), arsenite-methylating bacteria (AsMB), dissimilatory sulfate-reducing bacteria (DSRB), sulfur-oxidizing bacteria (SOB), dissimilatory Fe(III)-reducing bacteria (DFeRB), Fe(II)-oxidizing bacteria (FeOB)] was evaluated according to a reference database of microbial genera retrieved according to the literature and to data available at the National Center for Biotechnology Information (NCBI, Supplementary Dataset 1). Methanogenic archaea (i.e. MA) were retrieved using the PhyMET^2^ database (http://phymet2.biotech.uni.wroc.pl/, Burdukiewicz et al. 2018). The R-based package Tax4Fun2 (Wemheuer et al. 2020) was used to infer the presence of genes related to arsenic, sulfur and iron metabolisms, as well as to methanogenesis and methanotrophy.

### Quantification of microorganisms involved in arsenic, sulfur and iron transformations by RT-qPCR

To further analyze microorganisms putatively involved in arsenic cycling in rice rhizosphere and in pore water, the 16S rRNA genes of total bacteria and archaea and genes encoding the A subunit of arsenite oxidase (*aioA*), arsenate reductase (*arsC*), the A subunit of dissimilatory arsenate reductase (*arrA*), arsenite methyltransferase (*arsM*) and the A subunit of dissimilatory bisulfite reductase (*dsrA*) were amplified and quantified by real-time qPCR (RT-qPCR). Furthermore, 16S rRNA genes of the microorganisms belonging to iron-reducing *Geobacteriaceae* and *Shewanellaceae* and to iron-oxidizing *Gallionellaceae* were quantified. Details of primer pairs and protocols used in this study can be found in Supplementary Table 5. For each reaction, 10 ng of template DNA was mixed with primers and Titan HotTaq EvaGeen^®^ qPCR Mix (Bioatlas, Estonia), in a total volume of 20 µL. The thermal protocols were carried out on a QuantStudio^TM^ 3 System (Thermofisher, Waltham, MA, USA). The correct size of qPCR amplicons was checked by agarose gel electrophoresis. Standard curves were created by the amplification of the selected target from plasmid DNA (Supplementary Table 5). The abundance of the quantified functional genes was expressed as relative abundance by normalization to total bacterial and archaeal 16S rRNA genes, while the 16S rRNA genes of iron-cycling bacteria were normalized only to total bacterial 16S rRNA genes.

### Statistical analysis

The statistical analyses of Illumina 16S rRNA gene library data were performed using QIIME2 and the R program, v. 3.6.0 (R Core Team 2015), package vegan version 2.5–5 (Oksanen et al. 2020).

With the R base program, one-way analysis of variance (ANOVA), Tukey's b, Duncan and t-test at *P* ≤ 0.05 were used for comparisons in the analysis of the alpha diversity, of the abundance of microorganisms related to arsenic, sulfur and iron cycles and of the qPCR amplifications. The alpha diversity was analyzed by gathering the samples in different groups to evaluate the “compartment effect” (i.e. original unplanted soil vs rhizosphere soil vs pore water), the “soil type effect” (i.e. Fornazzo vs Veronica), the “sulfate amendment effect” (i.e. control vs sulfate) and the “time effect” (i.e. stem elongation vs flowering vs dough).

To compare bacterial and archaeal diversity among the samples, weighted UniFrac distances were calculated from rarefied ASV tables and principal coordinates analysis (PCoA) was performed (Lozupone et al. 2005, Hamady and Knight 2009, Halko et al. 2010). Significantly different groups of samples defined by the “compartment effect”, the “soil type effect”, the “sulfate amendment effect” and the “time effect” were identified, applying the permutational analysis of variance (PERMANOVA, permutations = 999), using the QIIME2 pipeline (Anderson 2001).

Significant differences in the abundance (i.e. differential abundance) of bacterial and archaeal families and genera retrieved with 16S rRNA genes Illumina sequencing due to the different soils and to sulfate application were tested using the quasi-likelihood F-test implemented in the R package EdgeR version 3.11 (Robinson et al. 2010, R Core Team 2015).

To highlight statistically significant positive and negative interactions among bacterial and archaeal genera, co-occurrence network analysis was performed by testing the probabilistic co-occurrence model on presence-absence genus tables using the R package cooccur version 1.3 (Veech 2013, Griffith et al. 2016). Positive and negative correlations were tested by grouping original unplanted soil, rhizosphere soil and pore water samples collected according to the compartment (i.e. original unplanted soil vs rhizosphere soil vs pore water), soil type (i.e. Fornazzo vs Veronica), sulfate amendment (i.e. control vs sulfate) and timing (i.e. stem elongation vs flowering vs dough). Co-occurrence analysis is based on the presence/absence of each genus in the samples. The genera that were not present in all the replicates of at least one sample with at least 20 reads were removed from the analysis. To estimate the number of possible keystone genera, each network was recalculated by removing one genus and calculating the percentage of lost connections without that genus. This process was repeated for all genera.

To investigate links between chemistry and microbial populations involved in arsenic, sulfur and iron cycles, linear Pearson correlations were calculated between the relative abundance of the different microbial populations in the rhizosphere soil and in the pore water and pore water physicochemical parameters [i.e. total arsenic, ferrous iron, total sulfur, methylated arsenic, methylated oxyarsenates, total thioarsenates, inorganic thioarsenates, methylated thioarsenates, total organic carbon (TOC), total inorganic carbon (TIC), pH and Eh] at each time point.

Possible statistically significant correlations between the bacterial and archaeal community compositions, the functional predictions, the physicochemical parameters measured in the pore water (i.e. total arsenic, ferrous iron, total sulfur, methylated arsenic, methylated oxyarsenates, total thioarsenates, inorganic thioarsenates, methylated thioarsenates, TOC, TIC, pH and Eh) and qPCR data were evaluated by applying redundancy analysis (RDA; Legendre and Legendre 2012) and the Mantel test (permutations = 999), both implemented in the vegan package (Legendre and Legendre 2012). Bacterial and archaeal genera abundance data, the relative abundance of microorganisms involved in arsenic, sulfur and iron cycles (as indicated in Supplementary Dataset 1) and the relative abundance of enzymes involved in arsenic, sulfur and iron cycles (as indicated in Supplementary Datasets 2 and 3) were Hellinger-transformed to calculate Bray–Curtis dissimilarities, while the physicochemical and qPCR data were log-transformed to calculate Euclidean dissimilarities (Legendre and Gallager 2001).

## Results

### Diversity of bacterial and archaeal communities

Illumina sequencing of 16S rRNA genes produced in total 330 206 and 551 058 high quality bacterial and archaeal reads, respectively (Supplementary Table 6). On average, the rhizosphere soil showed a higher number of ASVs than the pore water. This difference was more pronounced in the bacterial vs archaeal library and more pronounced in the higher organic C soil Fornazzo vs the lower organic C soil Veronica (Supplementary Table 6). Accordingly, bacterial and archaeal 16S rRNA genes biomarkers were higher in the higher organic C soil in both rhizosphere soil and pore water (data not shown).

**Figure 1.**
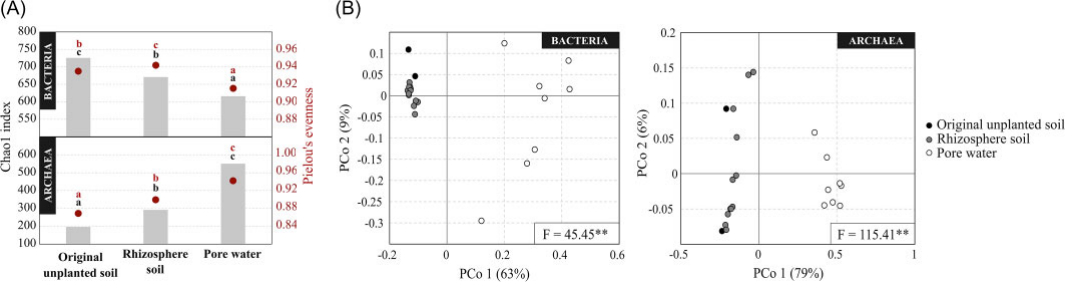
Alpha and beta diversity by means of Chao1 index and Pielou's evenness (A) and of weighted UniFrac dissimilarities of bacterial and archaeal communities in the original unplanted soil, rhizosphere soil and pore water. Comparisons were statistically tested to evaluate the compartment effect according to ANOVA (A; letters indicate significantly different groups; *P ≤ 0.05) and to the PERMANOVA test F (B; ** *P* ≤ 0.01).

Both bacterial species richness (Chao1 index) and evenness were significantly lower in the pore water with respect to the original unplanted soil, while an opposite trend was observed for Archaea, which were significantly richer and more uniform in the pore water samples compared with the original unplanted soil and with the rhizosphere soil (Fig. 1A, *P* ≤ 0.05). A soil type effect was observed for both Bacteria and Archaea in all compartments, each following different patterns (Supplementary Figure 1A, *P* ≤ 0.05). Archaeal Chao1 index negatively responded to sulfate amendment, being lower in all rhizosphere soil and pore water samples where sulfate was supplied, compared with the controls (Supplementary Figure 1B, *P* ≤ 0.001). In the rhizosphere soil, both bacterial and archaeal Chao1 index significantly decreased, while in the pore water the trend was more variable (Supplementary Figure 1C, *P* ≤ 0.05).

PCoA analysis based on weighted UniFrac revealed a significant “compartment effect” in both bacterial and archaeal communities (*P* ≤ 0.01, Fig. 1B). When analyzing the beta diversity dividing soil (i.e. original unplanted soil and rhizosphere soil) and pore water samples, a significant “soil type effect” was observed in both bacterial and archaeal communities in all compartments, while sulfate amendment and time effects were significant only in soil samples (*P* ≤ 0.05, Supplementary Figure 2A and B).

### Composition of rice rhizosphere bacterial and archaeal communities

**Figure 2.**
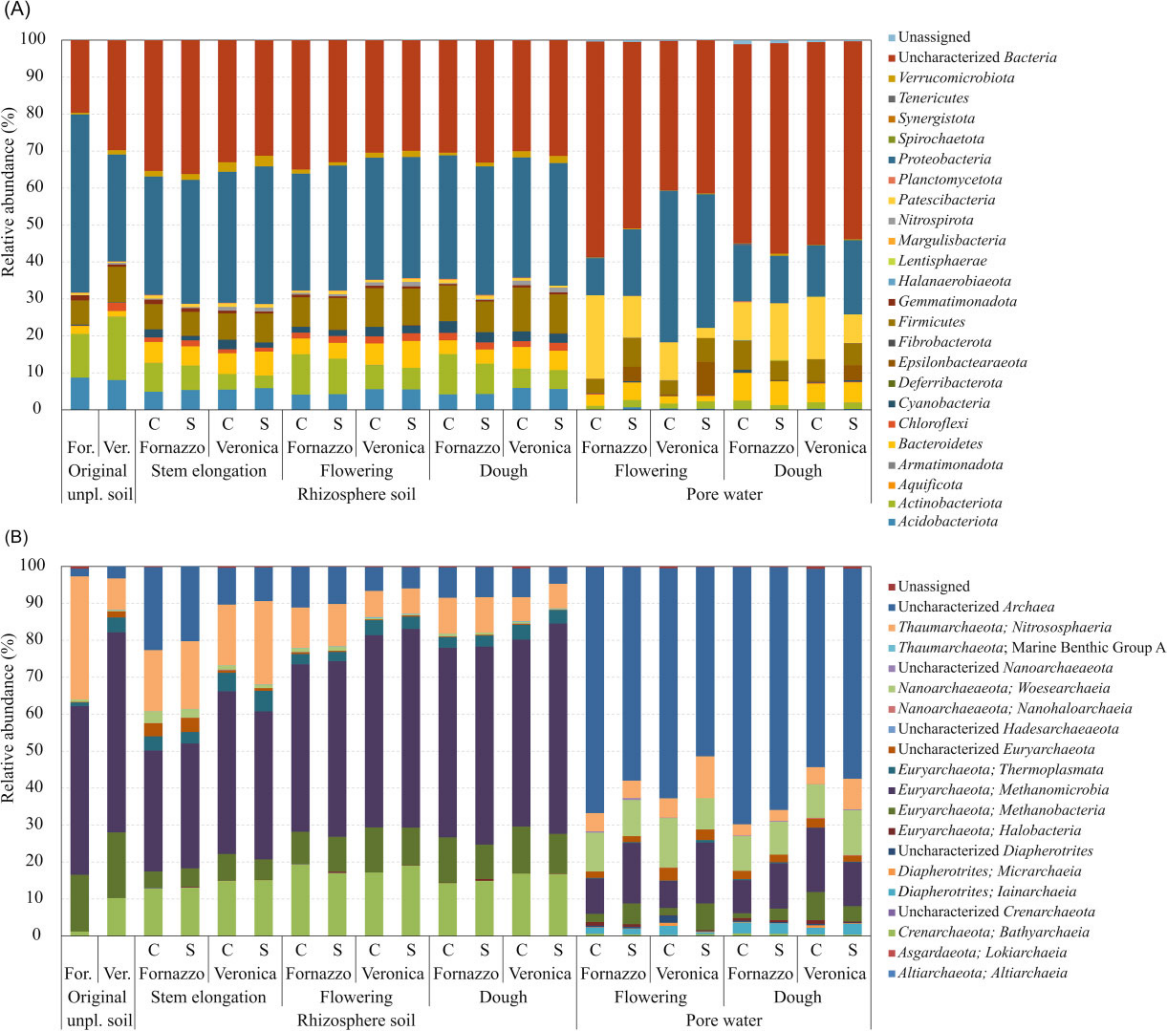
Relative abundance of bacterial (A) and archaeal (B) ASVs at phylum and class levels, respectively, retrieved in original unplanted soil, rhizosphere soil and pore water from Fornazzo and Veronica soils without (C) and with (S) sulfate amendment, at different time points (before seeding, stem elongation, flowering and dough).

Soil and pore water samples showed highly different composition in both bacterial and archaeal communities, evidencing a strong compartment effect (Fig. 2). In soil samples, the predominant bacterial phyla were *Proteobacteria, Actinobacteriota* (formerly *Actinobacteria*)*, Firmicutes, Acidobacteriota* (formerly *Acidobacteria*) and other uncharacterized *Bacteria* (relative abundance 20–30%; Fig. 2A). In the pore water, uncharacterized *Bacteria* (relative abundance 40–60%), *Proteobacteria* and *Patescibacteria* were the most abundant, and sulfate amendment increased the relative abundance of *Epsylonproteobacteraeota* (former class *Epsilonproteobacteria*) with the concomitant decrease of *Patescibacteria*. The compartment effect was evident also within *Proteobacteria*, being more abundant in soil samples, with the exception of *Gammaproteobacteria* that had their highest abundance in the pore water of Veronica soil (Supplementary Figure 3).

Concerning the archaeal communities, soil samples were dominated by *Euryarchaeota* (>70%, including *Methanomicrobia* and *Methanobacteria*), followed by *Crenarchaeota* (i.e. *Bathyarchaeia*) and *Thaumarchaeota* (i.e. *Nitrososphaeria*, Fig. 2B). In the pore water, uncharacterized archaeal phyla were dominant (relative abundance 50–70%), followed by *Methanomicrobia, Woesearchaeia* and *Nitrososphaeria*.

**Figure 3.**
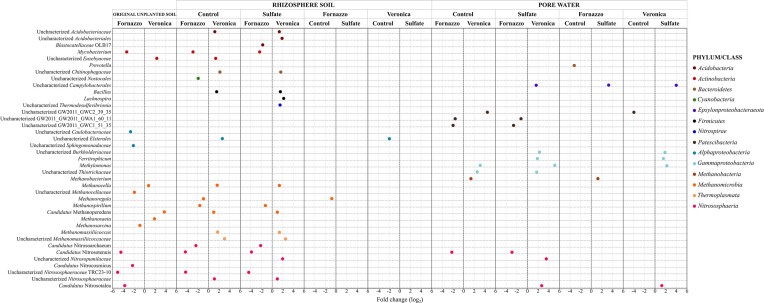
Bacterial and archaeal ASVs classified at genus level that significantly responded to soil type and sulfate amendment in the original unplanted soil, in rhizosphere soil and in pore water samples at the flowering time point (*P* ≤ 0.05).

Differential abundance analysis was performed at the genus level in all compartments at the flowering stage to evaluate the “soil type effect” and the “sulfate amendment effect”. In soil and pore water samples, different genera were significantly affected by the soil type and sulfate fertilization (Fig. 3). Most of the genera [i.e. 35 genera belonging to the *Acidobacteriota, Actinobacteriota, Bacteroidota* (formerly *Bacteroidetes*), *Cyanobacteria, Firmicutes, Nitrospirota* (formerly *Nitrospirae*), *Alphaproteobacteria, Gammaproteobacteria, Methanomicrobia, Thermoplasmata* and *Nitrososphaeria*] were significantly driven by soil type rather than by sulfate fertilization (Fig. 3, *P* ≤ 0.05). Sulfate fertilization significantly decreased the abundance of uncharacterized *Elsterales* in Veronica and of *Methanoregula* in Fornazzo in the rhizosphere soil, while in the pore water uncharacterized *Campylobacterales* and *Burkholderiaceae, Ferritrophicum, Methylomonas, Methanobacterium* and *Candidatus* Nitrosotalea significantly increased in sulfate-amended samples (*P* ≤ 0.05).

**Figure 4.**
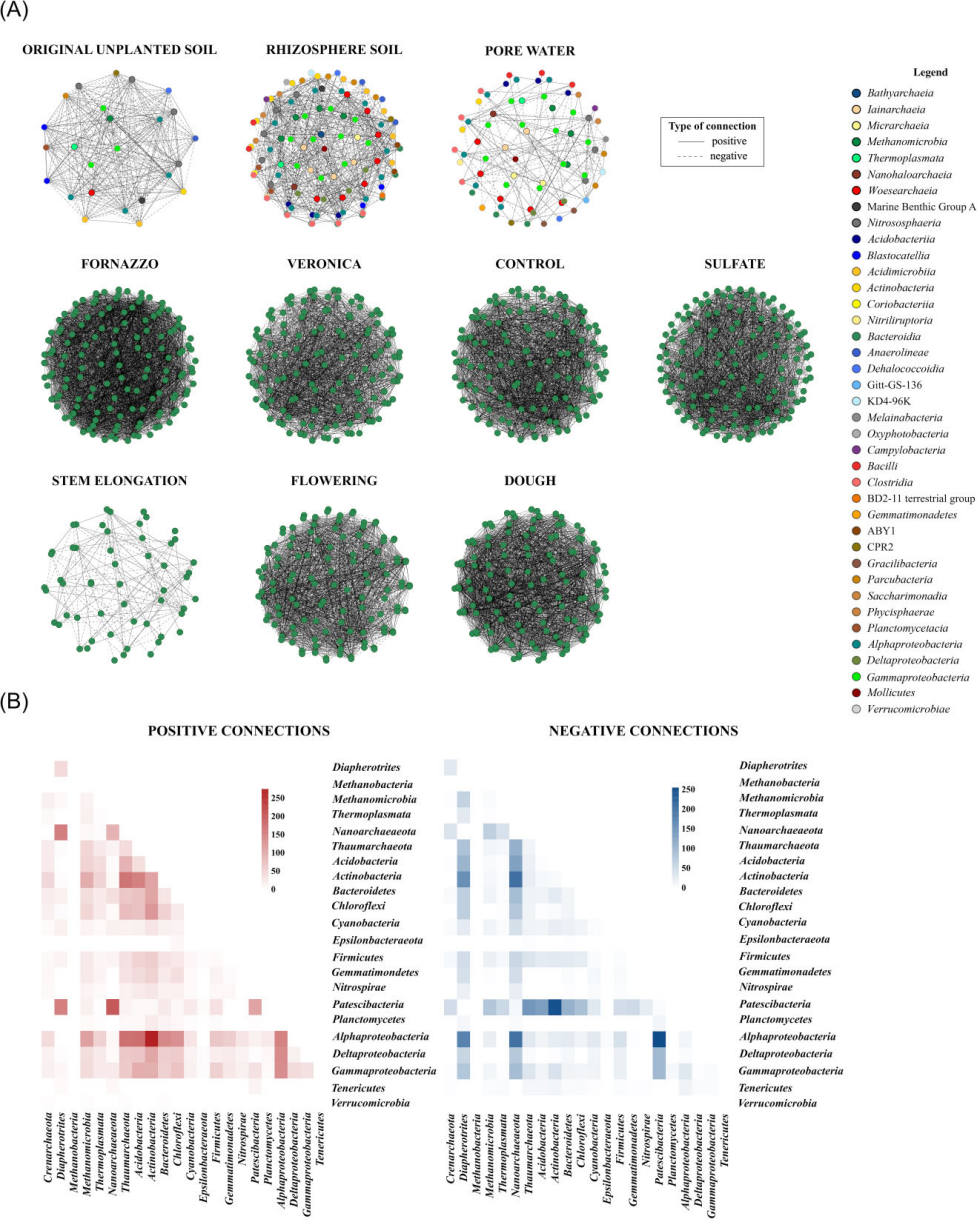
Co-occurrence network analysis of original unplanted soil, rhizosphere soil and pore water samples. Significant positive and negative correlations (*P* ≤ 0.05) were investigated by grouping the samples according to the compartment (original unplanted soil vs rhizosphere soil vs pore water), soil type (Fornazzo vs Veronica), sulfate amendment (control vs sulfate) and timing (stem elongation vs flowering vs dough), resulting in six different networks (A). The number of positive connections between classes/phyla were investigated to highlight possible syntrophic or antagonistic interactions, as well as the establishment of ecological niches (B).

Co-occurrence network analysis (Fig. 4) revealed that the compartment, the soil type, sulfate amendment and timing significantly affected specific correlations between microbial genera. In fact, (1) the number of connections was higher in unplanted original soil and in rhizosphere soil than in pore water (i.e. “compartment effect”), (2) a higher number of nodes and connections were present in lower C Veronica soil than in higher C Fornazzo soil (i.e. “soil type effect”), (3) less connections were observed in sulfate-amended samples (i.e. “sulfate amendment effect”) and (4) the number of connections increased over time (i.e. “time effect”) (Fig. 4A, Supplementary Dataset 4).

To evaluate the presence of possible keystone genera, data concerning the number of connections that were lost when each genus was removed (results shown in Supplementary Dataset 5) were compared with the number of genera that show a high number of connections (Supplementary Figure 4), with the proportion of genera responsible for the loss of at least one connection and the percentage of the maximum number of lost connections (Supplementary Figure 5). The networks based on the rhizosphere soil, the pore water and the stem elongation showed the lowest proportion of genera responsible for the loss of at least one connection (Supplementary Figure 5), suggesting the presence of a lower number of potential keystone genera in these samples if compared with the others. However, in the networks with the highest number of nodes and connections (i.e. Fornazzo, Veronica, control, sulfate, flowering and dough), the maximum number of lost connections from genera removal is lower with respect to the other networks. This might indicate that the trophic networks and the ecological niches in the samples characterized by the same soil type and by the same fertilization type and the ones established at the flowering and dough stages are likely more stable, probably because the same functions can be completed by different microbial genera. Therefore, the loss of one genus does not compromise the presence of a specific microbial function in the ecosystem, due to functional redundancy.

The highest number of positive and negative connections was observed between *Alphaproteobacteria, Patescibacteria* and *Actinobacteriota*, followed by *Thaumarchaeota, Acidobacteriota, Bacteroidota* and *Chloroflexi* (Fig. 4B). The genera with the highest number of positive connections were mostly affiliated to archaeal phyla, such as *Thaumarchaeota, Euryarchaeota* and *Crenarchaeota*, together with uncharacterized members of the *Acidobacteriota* family *Blastocatellaceae* (Supplementary Dataset 6). *Nitrospirota* showed the highest number of connections in proportion to the number of genera present in the phylum (Supplementary Figure 6), explained by the presence of only one uncharacterized genus within the order *Thermodesulfovibrionia*, significantly positively related to *Proteobacteria* genera (i.e. *Rhizobiales* genera, *Myxococcales* genera, *Sphingomonas, Comamonas, Desulfobacterium* and *Acinetobacter*).

In the sulfate-amended samples, a lower number of connections was mostly ascribable to a lower number of connections related to all archaeal phyla, concomitant to a higher number of connections of the genera *Desulfobacterium, Comamonas* and *Pseudomonas* when sulfate was applied (Supplementary Dataset 6).

### Inferred microbial functionalities and biomarkers related to arsenic, sulfur and iron biogeochemical cycles

Microbial functionalities related to arsenic, sulfur and iron cycles in the analyzed samples were inferred on the basis of the genera retrieved with Illumina sequencing of 16S rRNA genes (Supplementary Dataset 1).

**Figure 5.**
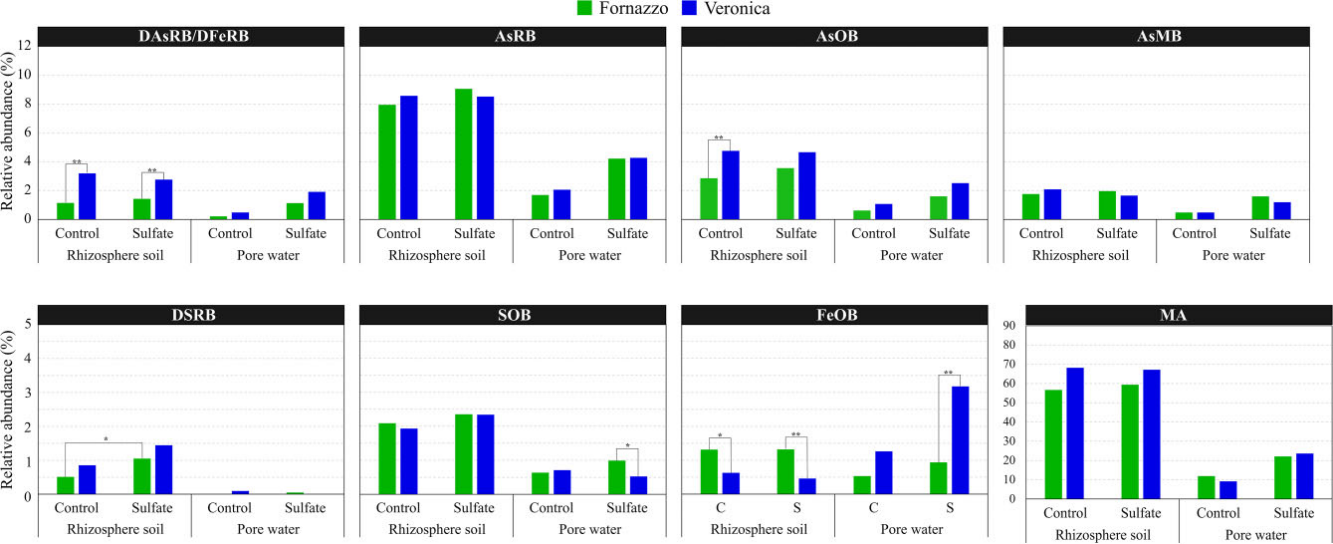
Relative abundance of genera putatively involved in arsenic, sulfur and iron transformation, and in methanogenesis in rhizosphere soil and pore water samples collected at the flowering time point. The genera included in this analysis were selected according to the literature, as well as to the presence of functional marker genes in their genome deposited in NCBI, as cited in Supplementary Dataset 1.

All the retrieved genera involved in arsenic, sulfur and iron cycles were in general more abundant in the rhizosphere soil than in the pore water (Supplementary Figure 7A), indicating that microbial populations of this compartment contribute mostly to those elemental cycling. These outcomes suggest that the compartment was the strongest driver if compared with soil type, sulfate amendment and timing. A significant effect was exerted by the soil type on DAsRB/DFeRB and on AsOB in the rhizosphere soil, and on pore water SOB and FeOB (Fig. 5). The two versatile genera *Bacillus* and *Geothermobacter* able to perform dissimilatory respiration of both arsenate and ferric iron were the only contributors to the group DAsRB/DFeRB (Supplementary Dataset 1).

Within sulfur cycling, a higher number of SOB was revealed (i.e. *Bacillus, Acidiphilium, Azospirillum, Methylobacterium, Bradyrhizobium, Rhodopseudomonas, Paracoccus, Acidithiobacillus, Comamonas* and *Polaromonas*) with respect to DSRB (Supplementary Figure 6, Supplementary Dataset 1). A “sulfate amendment effect” was only observed for DSRB (i.e. unclassified *Thermodesulfovibronia, Desulfobacterium, Desulfovibrio* and unclassified *Desulfobulbaceae*), which were generally within the “rare biosphere” (i.e. relative abundance <1%), although significantly more abundant in sulfate-amended rhizosphere soil samples (Fig. 5).

Some of the bacterial and archaeal genera that were involved in arsenic, sulfur and iron and methane cycles (i.e. included in Supplementary Dataset 1) were also involved in positive and/or negative correlations according to the co-occurrence analysis. These genera are highlighted in Supplementary Dataset 6. Specifically, MA belonging to the genera *Methanomassiliicoccus* and *Methanoculleus* showed more than 100 positive connections with other genera (Supplementary Dataset 6). A number of directly and indirectly arsenic-cycling bacterial genera showed a high number of positive connections, with *Clostridium, Mesorhizobium, Bradyrhizobium*, uncharacterized *Thermodesulfovibrionia, Desulfobacterium* and *Comamonas* among the most connected ones (Supplementary Dataset 6).

To implement the information on microbial functions inferred by the presence of specific microbial genera in the 16S rRNA gene library, predicted enzymes were investigated by Tax4Fun2 and specific gene biomarkers were quantified by RT-qPCR at the flowering stage.

**Figure 6.**
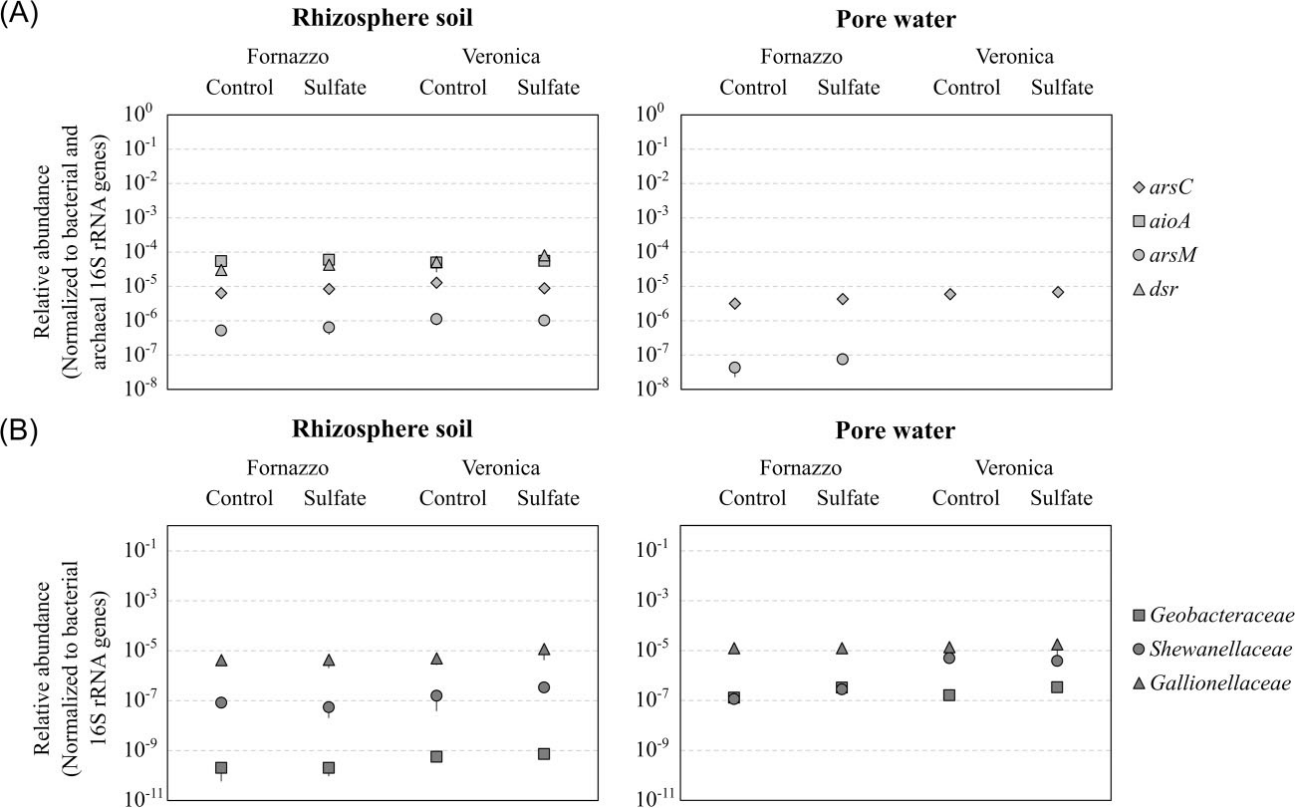
Relative abundance quantified by RT-qPCR of genes involved in arsenic and sulfur transformation (A) and of bacterial families involved in the iron cycle (B), in the rhizosphere soil and pore water of Fornazzo and Veronica soils, with and without sulfate amendment, at the flowering stage. Data of arsenic- and sulfur-transforming genes were normalized with respect to the total number of 16S rRNA gene copies of Bacteria and Archaea, while data of iron-cycling bacteria were normalized to total Bacteria only.

The ubiquitous arsenate detoxification system ARS (i.e. arsenate reductase ArsC, arsenite efflux pump ArsB) was detected in the pore water and in rhizosphere soil, where *arsC* was of the order of 10^4^ and 10^8^ copies per g of mL/dry soil, respectively, reflecting the ability of the Arsenate reductase to use soluble arsenic (Fig. 6, Supplementary Figure 7B). Arsenite oxidase coded by *aioA* gene was present only in rhizosphere soil at 10^8^ copies per g of dry soil, together with arsenite methyltransferase *arsM*, which was also retrieved in the pore water of higher organic C soil Fornazzo (Fig. 6, Supplementary Figure 7B). Here, the higher C content might have favored the presence of methylated groups. Genes encoding the respiratory arsenate reductase (i.e. ArrA) and the anaerobic arsenite oxidase (i.e. ArxA) were not retrieved by Tax4Fun2, nor by RT-qPCR, according to previous evidence in rice paddy soil from the same area (Zecchin et al. 2017a).

Sulfur cycle-related enzymes showed a compartment-dependent pattern, with sulfur oxidase (SoxAB) and thiosulfate reductase more abundant in rhizosphere soil samples, and enzymes involved in dissimilatory sulfate respiration (DsrAB) and respective biomarker *dsr* being absent in the pore water (Fig. 6, Supplementary Figure 7B). Sulfate amendment increased the abundance of enzymes involved in dissimilatory sulfate respiration (DsrAB) in the rhizosphere sample of the lower carbon soil Veronica, and SoxAB in the pore water (Fig. 6, Supplementary Figure 7B).

A “compartment effect” was observed for the relative abundance of most targets, with *aioA* and *dsr* only detected in rhizosphere soil samples, and *arsC* and *arsM* more abundant in the rhizosphere soil compared with the pore water (Fig. 6)

Regarding the iron cycle, DFeRB of the families *Geobacteraceae* and *Shewanellaceae* were more abundant in the pore water compared with the rhizosphere soil (Fig. 6), with *Shewanellaceae* significantly more abundant in Veronica samples (*P* ≤ 0.05). Sulfate amendment significantly increased the abundance of *Gallionellaceae* in Veronica rhizosphere soil, and of *Geobacteraceae* in both Fornazzo and Veronica, compared with the controls (*P* ≤ 0.05).

In general, microbial functions were more represented in the rhizosphere compartment with respect to the soil pore water. This habit reflects either the higher bacterial biodiversity that characterizes the soil compartment, and the fact that a large part of the pore water microbial community comprised uncharacterized ASVs. For such ASVs the prediction of functions might fail to give a correct picture.

### Correlation among microbial diversity, functionality and environmental parameters

**Figure 7.**
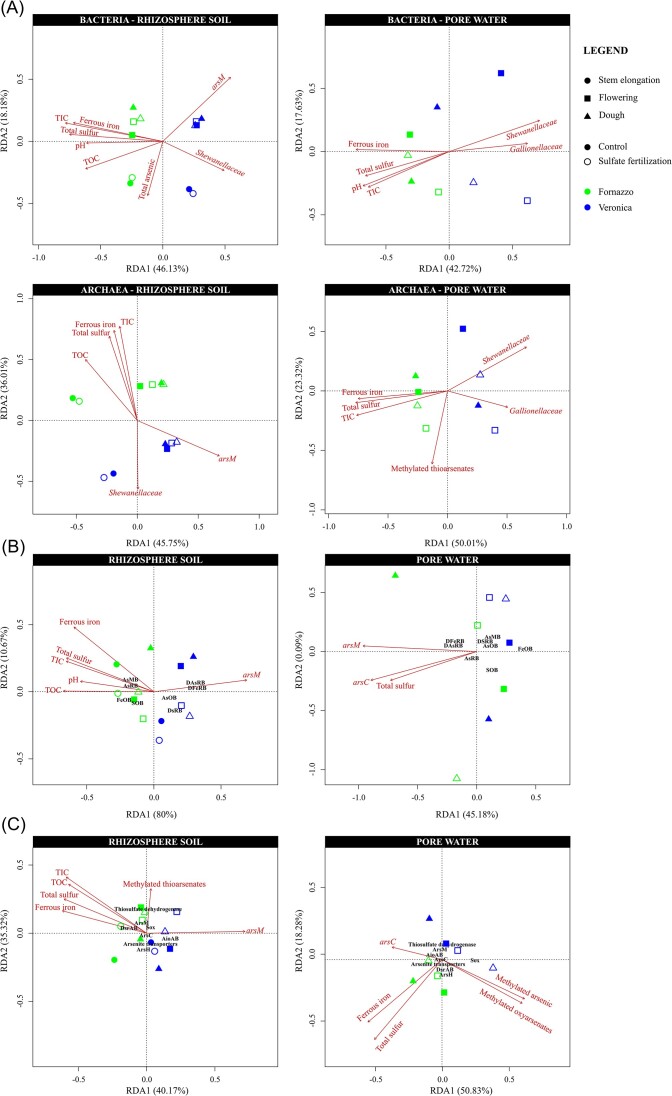
Redundancy analysis (RDA) of bacterial and archaeal communities in rhizosphere soil and pore water from Fornazzo and Veronica soils with and without sulfate amendment (A), of the distribution in the samples of microbial populations involved in arsenic, iron and sulfur cycles according to dataset 1 (B), and of the distribution of the functionalities involved in arsenic iron and sulfur cycles according to Tax4Fun2 analysis (C). Red arrows indicate physicochemical parameters and genes quantified with RT-qPCR that are significantly correlated with the microbial community composition of the samples, according to the Mantel test.

The correlation among the microbial community composition and functionality in the samples and the main physicochemical parameters measured in the porewater (i.e. total arsenic, total sulfur, ferrous iron, total thioarsenates, inorganic thioarsenates, methylated thioarsenates, methylated oxyarsenates, TOC, TIC, pH and Eh), as well as the qPCR quantifications, were investigated by RDA analysis (Fig. 7) and the Mantel test (Supplementary Tables 7, 8 and 9).

In both the rhizosphere soil and the porewater, the beta diversity of bacterial and archaeal communities was significantly driven by TIC, ferrous iron and total sulfur concentrations (all higher in Fornazzo samples; *P* ≤ 0.05, Fig. 7A, Supplementary Table 7). TOC significantly shaped the bacterial and archaeal communities in the rhizosphere soil (*P* ≤ 0.05, Fig. 7A, Supplementary Table 7). pH was significantly correlated with the bacterial communities in both the rhizosphere soil and the pore water (*P* ≤ 0.05, Fig. 7A, Supplementary Table 7). In the rhizosphere soil, the bacterial community was significantly shaped by total arsenic concentration (*P* ≤ 0.05, Fig. 7A, Supplementary Table 7). On the other hand, the archaeal community living in the pore water was significantly related to the concentration of methylated thioarsenates (*P* ≤ 0.05, Fig. 7A, Supplementary Table 7). *Shewanellaceae* 16S rRNA and *arsM* genes quantified by qPCR were significantly related to the beta diversity of both bacterial and archaeal communities in both the rhizosphere soil and pore water samples, being more abundant in Veronica samples (*P* ≤ 0.05, Fig. 7A, Supplementary Table 7). In the pore water, both bacterial and archaeal communities were significantly related to *Gallionellaceae* 16S gene copy number that was higher in Veronica samples (*P* ≤ 0.05, Fig. 7A, Supplementary Table 7).

The RDA between inferred functionalities and physico-chemical parameters showed a similar pattern in the rhizosphere soil, were the abundance of microbial species (i.e. DAsRB/DFeRB, AsRB, AsOB, AsMB, FeOB and SOB) and enzymes (ArsC, ArsB, ArsM, ArsH, DsrAB and Sox) involved in arsenic, sulfur and iron cycles were shaped by TIC, TOC, total sulfur and ferrous iron and were related to *arsM* gene copy number (*P* ≤ 0.05, Fig. 7B and C, Supplementary Tables 8 and 9). On the other hand, the distribution of the specific genera in the pore water samples was shaped by total sulfur and significantly related to *arsC* and *arsM* gene copies (*P* ≤ 0.05, Fig. 7B, Supplementary Table 8), while Tax4Fun2-inferred enzymes were significantly related to total sulfur and ferrous iron, as well as to the concentration of methylated arsenic (*P* ≤ 0.05, Fig. 7C, Supplementary Table 9).

To further investigate whether the dynamics in the microbial populations observed in this study support previously reported differences in chemistry between the two soils, Pearson linear correlation tests were performed between different pore water chemistry parameters (i.e. total arsenic, total sulfur, total thioarsenates, inorganic thioarsenates, methylated thioarsenates, methylated oxyarsenates, TOC, TIC, pH and Eh) and rhizospheric and pore water arsenic-, sulfur- and iron-cycling microbial populations.

While arsenic did not show any correlation with microbial populations involved in arsenic, sulfur and iron cycles, Fe(II), total S, TIC and TOC were significantly correlated with different microbial populations, showing different trends (Supplementary Table 10). Specifically, Fe(II) was negatively correlated with rhizospheric AsOB and DSRB, while total S, TIC and TOC were negatively correlated with rhizospheric DAsRB/DFeRB, AsOB and DSRB but positively correlated with rhizospheric FeOB. Interestingly, rhizospheric SOB were positively correlated with different thiolated and methylated As species (i.e. total thioarsenates, inorganic thioarsenates, methylated thioarsenates). Pore water FeOB were negatively correlated with Fe(II) and total S, but positively correlated with methylated arsenic. Rhizospheric DAsRB/DFeRB, AsOB and DSRB, and pore water FeOB, are significantly negatively driven by Fe(II) and total S, which are lower in Veronica soil. This outcome is in accordance with previously shown data (in Fig. 5), where these populations were more abundant in Veronica soil. Moreover, these data suggest a link between SOB and arsenic thiolation and between FeOB and methylated arsenic.

## Discussion

To the best of our knowledge, the analyses performed in the present study revealed for the first time that paddy field pore water harbors specific microbial populations that are distinct from the ones inhabiting the original unplanted soil and the rhizosphere soil, thus confirming that the compartment, likely characterized by different physico-chemical properties, is the major driver in shaping these different ecosystems. In fact, previous studies on rice paddy soil compartments were only focused on unplanted, bulk or rhizosphere soil, and demonstrated the existence of a “rhizosphere effect” due to the presence of a high amount of root exudates coupled to O_2_ leaking from root aerenchyma that together fuel microbial organic matter degradation, respiration and fermentation processes (Lynch and Whipps 1990, Revsbech et al. 1999, Liesack et al. 2000, Demoling et al. 2007, Marschner 2011, Wörner et al. 2016, Huaidong et al. 2017, Ding et al. 2019).

Unexpectedly, archaeal diversity was found to be higher in the pore water with respect to the original unplanted soil and to the rhizosphere soil. While a sharp separation of the rhizosphere soil samples from the bulk soil was ensured by the protocol followed (in accordance with Zecchin et al. 2017b), we cannot ensure that the sampled pore water was derived exclusively from the rhizosphere area. Hence, in the pore water samples, microorganisms deriving from the anoxic bulk soil area were likely included. One hypothesis could be that a proportionally higher number of anaerobic and oligotrophic archaeal species are present in the pore water compartment compared with the bacterial community. This possible explanation might be found in the lifestyle of pore water archaeal species, which is, however, still poorly inferable because most of the retrieved genera were uncharacterized. These outcomes suggest the importance of further investigating microbial communities in rice paddy pore water in order to clarify the role of uncharacterized microbial taxa in element cycling.

While the rhizosphere soil was dominated by *Proteobacteria, Acidobacteriota, Actinobacteriota* and methanogenic Archaea typically found in the rice rhizosphere (Bao et al. 2016, Ding et al. 2019), the pore water microbial communities were more related to aquatic ecosystems, and mostly hosted uncharacterized microbial species. Among these, members of the phylum *Patescibacteria*, including microbial taxa formerly assigned to the “Candidate Phyla Radiation” (CPR) group (Parks et al. 2018), were dominant. These microorganisms are widely distributed in aquatic subsurface environments, where they were suggested to have a fermentative lifestyle and to be associated with autotrophic iron- and sulfur-cycling microorganisms (Herrmann et al. 2019). The pore water archaeal community hosted a significantly higher proportion of members of the DPANN archaeal superphylum (i.e. *Diapherotrites, Parvarchaeota, Aenigmarchaeota, Nanoarchaeota* and *Nanohaloarchaeota*) compared with the rhizosphere soil. This superphylum includes a variety of still poorly characterized small-sized microorganisms with diverse metabolic features that are supposed to be widespread in the environment (Moissl-Eichinger et al. 2018). The presence of DPANN in agricultural soils, including rice paddies, was recently reported, however, without further discussion (Wan et al. 2021, Cho et al. 2022). Members of the phylum *Nanoarchaeota* include putative sulfide-oxidizers that live as obligate endosymbionts of *Crenarchaeota* (St. John et al. 2019), and might play a crucial role in sulfur cycling in rice paddies and in plant detoxification from reduced sulfur compounds.

Interestingly, both Illumina sequencing and qPCR indicated that the relative abundance of most of the microorganisms involved in arsenic, sulfur and iron cycles were in general more abundant in the rhizosphere soil compared with the pore water, suggesting that organic matter, the surface of soil particles and minerals have a crucial role in mediating microbial reactions in rice field soil, thus influencing the element biogeochemical cycles as reported before (Hoffman et al. 2021, Crundwell 2013). However, because several uncharacterized bacterial and archaeal genera were retrieved in the pore water, the relative importance of pore water microbial communities with respect to rhizosphere soil in element cycling should be confirmed by further investigations.

Overall, the results underline that the “soil type” is defined by a complex of physico-chemical parameters (i.e. mainly organic C content, iron and sulfur) that were the main drivers in the taxonomic and functional shaping of the microbial communities, rather than sulfate addition. In fact, within the strong influence determined by the compartment, the two soils were originally characterized by distinct microbial communities, with differentially abundant bacterial and archaeal genera, and over time the differentiation was maintained both in the composition and in the ecological networks despite a similar agronomic management. Moreover, the soil type strictly defined specific physicochemical parameters (organic and inorganic C, iron, sulfur and pH) that selected specific microbial populations directly or indirectly involved in arsenic cycling. This confirms the relevance of pore water C content and redox potential in shaping the rhizosphere microbial populations involved in arsenic biogeochemistry in rice paddies, as hypothesized in previous studies (Somenahally et al. 2011, Zecchin et al. 2017a, Yang et al. 2018, Ma et al. 2014, 2020, Dai et al. 2020, Hossain et al. 2021). Microbial communities in the rhizosphere soil and in the pore water were differently influenced by pore water C. In fact, while pore water TIC and TOC were significantly related to both the phylogenetic and functional diversity of the rhizosphere microbial communities, these parameters were only weakly or not related to the pore water microbiome. The presence of several uncharacterized microbial genera in the pore water might have biased these outcomes. In this regard, the soil type had a certain but rather minor role. In fact, the lower organic C content in Veronica soil corresponded to a lower microbial proliferation in comparison with Fornazzo soil, as demonstrated in the present study by absolute qPCR quantification, but this difference was not reflected by a lower number of microbial species. This might indicate that not only the concentration but also the quality of organic C substrates inherited from the soil is important for microbial community shaping. Some works suggest that the soluble C released from the added rice straw is rapidly utilized, supplying easily degradable electron donors that may prime microbially catalyzed reductive dissolution of soil iron minerals; however, it is the progressive release of previously iron-stabilized organic C that feeds the microbial communities during the whole growing season (Said-Pullicino et al. 2016; Ye and Howrath, 2017).

The outcomes from the present study showed that sulfate amendment suppressed several positive and negative correlations driven by archaeal genera, probably not only in relation to sulfate, but to a general higher nutrient availability in rice rhizosphere in comparison with the unamended mesocosms. This aspect also emerged in the study of Liu et al. (2021), where alternating wet–dry cycles were found to be more efficient than sulfate fertilization in decreasing CH_4_ production in Veronica straw-amended soils. The hypothesis is that in soils with high C content, sulfate fertilization might not be crucial for DSRB to outcompete MA.

In the present study, sulfate amendment increased the relative abundance of DSRB, as previously observed by Wörner et al. (2016), and SOB. Many genera that positively responded to sulfate amendment were uncharacterized bacteria and archaea. For some of these, the presence of sulfur cycling as a crucial metabolic trait was inferred by previous in vivo studies, as for the DSRB class *Thermodesulfovibronia* (i.e. phylum *Nitrospirota*; Sekiguchi et al. 2008, Anantharaman et al. 2016, Zecchin et al. 2018, Umezawa et al. 2020, 2021, Arshad et al. 2017, 2018, Kato et al. 2018) and for the SOB *Campylobacterales* (Inagaki et al. 2003, 2004, Kodama and Watanabe 2004, Sievert et al. 2007, Tan and Foght 2014, Stolz et al. 2015). These observations, coupled to a relatively high abundance of SOB, support previous hypotheses that sulfur cycling occurs at high rates in rice rhizosphere, and that it is stimulated by sulfate amendment (Pester et al. 2012, Wörner et al. 2016, Zecchin et al. 2018). In rice paddies, DSRB might have a crucial role in arsenic thiolation by the production of sulfide, as previously observed for *Desulfovibrio desulfuricans* in the human gut (DC. Rubin et al. 2014).

In the pore water of lower organic C Veronica soil, the obligate FeOB *Ferritrophicum*, responsible for iron plaque formation in wetland plants (Weiss et al. 2007), positively responded to sulfate fertilization. The significant increase of these iron-related microorganisms in sulfate-amended rice paddy mesocosms in the lower organic C soil contributes to explain the effect of sulfate amendment in decreasing dissolved arsenic. Initially, this low organic C soil already had a significantly higher abundance of DAsRB/DFeRB and DSRB in the rhizosphere soil and of FeOB in the pore water than in the high organic C soil. So, the more marked effect of sulfate amendment observed in Veronica pore water in decreasing dissolved arsenic with respect to Fornazzo might be explained (1) by a higher immobilization of arsenic with enhanced iron plaque production by FeOB, similar to what was previously described by Hu et al. (2007); and/or (2) by a higher co-precipitation or adsorption of arsenite (produced by DAsRB), with secondary iron sulfide minerals (produced by DFeRB and DSRB), or rather, as suggested before (Wang et al. 2020b), by mixed Fe(II)Fe(III) minerals (produced by DFeRB coupled to re-oxidation of reduced sulfur). Moreover, the significant positive correlation between SOB and total sulfur might contribute to explain the higher thiolation of arsenic in Veronica pore water with respect to Fornazzo, providing locally higher concentrations of sulfide for arsenic thiolation and supporting a more active sulfur cycling. It might be hypothesized that all three proposed microbial processes might have occurred in the mesocosms, resulting in the lower total arsenic mobility and higher percentage contribution to total arsenic of thioarsenates in Veronica compared with Fornazzo. In the lower carbon soil, the presence of SOB and DSRB supports an active sulfur cycle fueled by available sulfur species. The positive correlation between SOB and thiolated arsenic species might be explained by the availability of S^0^ and SO_4_^2−^ to be used as electron acceptors by DSRB that produce sulfide and increment thiolation. On the other hand, in higher carbon soil, the formation of FeS subtracts reduced sulfur from pore water thus establishing a less active microbially mediated sulfur cycle, therefore lowering thiolated arsenics.

In view of the upcoming water scarcity due to climate change, these outcomes suggest that a lower pore water content will probably have dramatic effects on element cycling mediated by microbial populations both present in the different soil/rhizosphere/water compartments and affected by the redox potential. It was previously shown (Zecchin et al. 2017a) that the microbial communities developed in the rhizosphere soil of rice cultivated under aerobic conditions were completely different if compared with the ones inhabiting the rhizosphere soil of continuously flooded rice plants. Hence, a lower water content in rice paddy soils is expected to progressively decrease the activity of pore water-specific microbial populations such as FeOB to a level that will depend on the extent of water scarcity and/or to the type of water management adopted in the different agronomic schemes. Moreover, because rhizospheric microbial communities are significantly shaped by the pore water parameters, a decrease of soil water content might in general slow down microbial arsenic, sulfur and iron cycling in rice paddies. This eventuality should be carefully considered by working on predictive models that include the outcomes of the present and all previous available data.

Overall, the data obtained in this study revealed that the compartment effect in rice paddy soil is the major driver of microbial diversity and functionality, ultimately affecting the complex interplay between microorganisms in rice paddy soil and arsenic, sulfur and iron mobility.

The microbial communities inhabiting the rhizosphere soil and the pore water developed over time were completely different and responded differently to sulfate amendment. In each compartment, soil type and C content significantly drove the development of the microbial communities. The effect of sulfate amendment was also compartment-specific. It was linked to iron and sulfur concentrations and, in the low-C soil, promoted iron-oxidizing bacteria, dissimilatory arsenate-, iron- and sulfate-reducing bacteria, which were likely responsible for arsenic sequestration by secondary iron minerals and/or iron sulfides and its subsequent decrease in the pore water. The higher proportion of arsenic thiolation measured in sulfate-amended compared with unamended soil was found to be related to dissimilatory arsenate-, sulfate-reducing bacteria and sulfur-oxidizing bacteria.

These aspects should be considered carefully in the future, when the need to face a progressive drought due to climate change will necessarily lead to the adoption of more water-saving agronomic schemes in rice cultivation.

## Supplementary Material

fiad121_Supplemental_File

## Data Availability

The raw reads obtained with Illumina sequencing of 16S rRNA genes were deposited in GenBank within the Bioproject PRJNA858795 and in the Dataverse repository (https://dataverse.unimi.it/dataverse/P-RICE).
