# Supplementary material for: Microbial communities in paddy soils: differences in abundance and functionality between rhizosphere and pore water, the influence of different soil organic carbon, sulfate fertilization and cultivation time, and contribution to arsenic mobility and speciation
Source: FEMS Microbiol Ecol. 2023 Oct 6;99(11):fiad121. doi: 10.1093/femsec/fiad121 (PMC10630088; doi:10.1093/femsec/fiad121)
Supplement: fiad121_Supplemental_File [file fiad121_supplemental_file.zip › Zecchin_et_al_Supplementary_Information_FEMS_ME_REVISED_supplementary_data.docx]

*Supplementary Information*

**Microbial communities in paddy soils: Differences in abundance and functionality between rhizosphere and pore water, influence of different soil organic carbon, sulfate fertilization, and cultivation time, and contribution to arsenic mobility and speciation**

Sarah Zecchin^a^, Jiajia Wang^b^, Maria Martin^c^, Marco Romani^d^, Britta Planer-Friedrich^b^, Lucia Cavalca^a^*

*^a^ Dipartimento di Scienze per gli Alimenti, la Nutrizione e l'Ambiente (DeFENS), Università degli Studi di Milano, Milano, Italy*

*^b^ Department of Environmental Geochemistry, Bayreuth Center for Ecology and Environmental Research (BAYCEER), Bayreuth University, Germany*

*^c^ Department of Agriculture, Forest and Food Science, University of Turin, Turin, Italy*

*^d^ Rice Research Centre, Ente Nazionale Risi, Castello d’Agogna, Pavia, Italy*

*Corresponding author. Dipartimento di Scienze per gli Alimenti, la Nutrizione e l'Ambiente (DeFENS), Università degli Studi di Milano, Milano, Italy.

E-mail address: lucia.cavalca@unimi.it.

**1. Supplementary tables**

**Supplementary Table 1** Physicochemical characterization of the soils used in this study (from Wang et al., 2020).

| **Parameters** | **Fornazzo** | **Veronica** |
| --- | --- | --- |
| Coordinates | E 8°57′50′′, N 45°13′54′′ | E 8°53′48″, N 45°10′39′′ |
| Parent material | Olocene alluvium | Pleistocene alluvium |
| pH | 5.8 | 5.6 |
| C (%) | 4.7 | 2.0 |
| C (mmol kg^-1^) | 3917 | 1667 |
| N (%) | 0.5 | 0.6 |
| Total As (mg kg^-1^) | 5.6 | 5.8 |
| Total S (mmol kg^-1^) | 100 | 81 |
| Fe* (mmol kg^-1^) | 71 | 52 |

*0.5 M HCl extractable Fe

**Supplementary Table 2** Chemical analyses performed on the pore waters sampled at the flowering stage (from Wang et al., 2020).

| **Soil** | **Treatment** | **Time point** | **TIC** | **TOC** | **Tot As** | **Tot S** | **Fe(II)** | **Eh** | **pH** |
| --- | --- | --- | --- | --- | --- | --- | --- | --- | --- |
|  |  |  | mg L^-1^ | mg L^-1^ | µg L^-1^ | mg L^-1^ | mmol L^-1^ | mV |  |
| Fornazzo | Control | Stem elongation | 88.92 ± 7.44 | 135.92 ± 21 | 22.02 ± 2.44 | 28.16 ± 2.00 | 0.43 ± 0.04 | 163 ± 15 | 7.3 ± 0.1 |
|  |  | Flowering | 100.94 ± 12.11 | 131.41 ± 49.74 | 19.65 ± 4.60 | 31.30 ± 5.03 | 0.43 ± 0.04 | 116 ± 19 | 7.1 ± 0.1 |
|  |  | Dough | 96.99 ± 17.99 | 96.37 ± 35.54 | 15.63 ± 3.34 | 34.21 ± 5.90 | 0.41 ± 0.06 | 130 ± 36 | 7.2 ± 0.2 |
|  | Sulfate | Stem elongation | 76.07 ± 3.13 | 116.33 ± 26.03 | 18.45 ± 1.01 | 24.38 ±0.90 | 0.40 ± 0.08 | 112 ± 50 | 7.1 ± 0.1 |
|  |  | Flowering | 89.25 ± 0.76 | 105.03 ± 10.82 | 15.51 ± 0.75 | 25.36 ± 0.44 | 0.37 ± 0.06 | 69 ± 10 | 7.1 ± 0.1 |
|  |  | Dough | 99.84 ± 5.25 | 104.26 ± 10.07 | 14.05 ± 0.41 | 26.85 ± 1.63 | 0.39 ±0.03 | 80 ± 11 | 7.5 ± 0.1 |
| Veronica | Control | Stem elongation | 19.46 ± 1.75 | 94.27 ± 25.89 | 41.19 ± 10.28 | 9.21 ± 0.61 | 0.25 ± 0.01 | 168 ±62 | 6.8 ± 0.1 |
|  |  | Flowering | 16.7 ± 2.62 | 91.58 ± 60.92 | 17.81 ± 4.89 | 11.2 ± 2.12 | 0.31 ± 0.07 | 122 ± 5 | 6.5 ± 0.1 |
|  |  | Dough | 15.81 ±5.37 | 64.89 ± 41.32 | 7.67 ±1.55 | 11.32 ± 3.60 | 0.34 ± 0.14 | 179 ± 19 | 6.8 ± 0.1 |
|  | Sulfate | Stem elongation | 15.41 ± 4.62 | 85.81 ± 32.97 | 11.19 ± 1.07 | 18.35 ± 13.56 | 0.27 ± 0.02 | 131 ± 36 | 6.9 ± 0.2 |
|  |  | Flowering | 25.44 ± 5.94 | 95.54 ± 47.79 | 10.20 ± 2.27 | 10.25 ± 1.81 | 0.28 ± 0.07 | 92 ± 16 | 6.8 ± 0.1 |
|  |  | Dough | 28.73 ± 3.37 | 94.95 ± 53.44 | 9.69 ± 4.03 | 10.39 ± 2.34 | 0.29 ± 0.07 | 149 ± 18 | 7.1 ± 0.1 |

**Supplementary Table 3** Percentage of total sulfur and ferrous iron mobilized to the pore water over time

| **Soil** | **Treatment** | **% mobilized total S** | **% mobilized Fe(II)** |
| --- | --- | --- | --- |
|  |  | (pore water S/soil S)*100 | (pore water Fe/soil Fe)*100 |
| Fornazzo | Control | 0.98 | 0.60 |
|  | Sulfate | 0.80 | 0.54 |
| Veronica | Control | 0.41 | 0.58 |
|  | Sulfate | 0.50 | 0.54 |

**Supplementary Table 4** Concentration of methylated and thiolated arsenic species in the pore water sampled at the flowering stage (from Wang et al., 2020).

| **Soil** | **Treatment** | **Stage** | **Methylated arsenic** | **Methylated oxyarsenates** | **Total thioarsenates** | **Inorganic thioarsenates** | **Methylated thioarsenates** |
| --- | --- | --- | --- | --- | --- | --- | --- |
|  |  |  | ppb | ppb | ppb | ppb | ppb |
| Fornazzo | Control | Stem elongation | 2.26 ± 0.83 | 2.02 ± 0.74 | 0.35 ± 0.09 | 0.10 ± 0.05 | 0.24 ± 0.13 |
|  |  | Flowering | 1.84 ± 0.51 | 1.60 ± 0.52 | 0.49 ± 0.17 | 0.25 ± 0.15 | 0.24 ± 0.02 |
|  |  | Dough | 0.90 ± 0.56 | 0.77 ± 0.55 | 0.44 ± 0.28 | 0.31 ± 0.29 | 0.13 ± 0.01 |
|  | Sulfate | Stem elongation | 1.15 ± 0.09 | 0.97 ± 0.10 | 0.66 ± 0.28 | 0.48 ± 0.27 | 0.18 ± 0.02 |
|  |  | Flowering | 1.11 ± 0.23 | 0.83 ± 0.18 | 0.91 ± 0.17 | 0.63 ± 0.21 | 0.28 ± 0.06 |
|  |  | Dough | 1.47 ± 1.29 | 1.26 ± 1.21 | 0.83 ± 0.11 | 0.61 ± 0.19 | 0.21 ± 0.08 |
| Veronica | Control | Stem elongation | 1.40 ± 1.04 | 1.37 ± 1.02 | 0.23 ± 0.22 | 0.20 ± 0.24 | 0.03 ± 0.03 |
|  |  | Flowering | 1.26 ± 1.12 | 1.24 ± 1.13 | 0.34 ± 0.16 | 0.33 ± 0.17 | 0.01 ± 0.01 |
|  |  | Dough | 0.44 ± 0.22 | 0.41 ± 0.17 | 0.23 ± 0.14 | 0.20 ± 0.17 | 0.03 ± 0.05 |
|  | Sulfate | Stem elongation | 1.28 ± 0.53 | 1.11 ± 0.27 | 0.69 ± 0.15 | 0.51 ± 0.16 | 0.17 ± 0.26 |
|  |  | Flowering | 2.28 ± 1.31 | 1.94 ± 1.28 | 0.60 ± 0.22 | 0.26 ± 0.10 | 0.34 ± 0.13 |
|  |  | Dough | 2.21 ± 1.83 | 2.08 ± 1.75 | 0.33 ± 0.10 | 0.20 ± 0.14 | 0.13 ± 0.09 |

**Supplementary Table 5** Primer pairs and reaction set ups used for qPCR quantifications.

| **Target** | **Primer** | **Sequence** | **Concentration (µM)** | **Annealing temperature (°C)** | **Product**  **size**  **(bp)** | **Standard DNA** | | **Reference** |
| --- | --- | --- | --- | --- | --- | --- | --- | --- |
| Total Bacteria | Eub338F | ACT CCT ACG GGA GGC AGC AG | 0.3 | 53 | 200 | | *Pseudomonas veronii* strain R02 | Fierer et al., 2005 |
|  | Eub518R | ATT ACC GCG GCT GCT GG |  |  |  |  |  |  |
| Total Archaea | Arc787F | ATT AGA TAC CCSB GTA GTC CAG GAA | 0.3 | 60 | 272 | | *Methanobacterium formicicum* | Yu et al., 2005 |
|  | Arc1059R | GCC ATG CAC CCW CCT CT |  |  |  |  |  |  |
| *aioA* | AoxBM1-2F | CCA CTT CTG CAT CGT GGG NTG YGG NTA | 0.3 | 59 | 550 | | *Achromobacter* sp. strain 1L | Quéméneur et al., 2008; Quéméneur et al., 2010 |
|  | AoxBM2-1R | GGA GTT GTA GGC GGG CCK RTT RTG DAT |  |  |  |  |  |  |
| *arsC* | ArsC52F | AGC CAA ATG GCA GAA GC | 0.4 | 55 | 275 | | *Arthrobacter* sp. CH72 | Bachate et al., 2009 |
|  | ArsC323R | GCT GGR TCR TCA AAT CCC CA |  |  |  |  |  |  |
| *arrA* | ArrAF | AAG GTG TAT GGA ATA AAG CGT TTG TBG GHG AYT T | 0.5 | 55 | 160-200 | | - | Malasarn et al., 2004 |
|  | ArrAR | CCT GTG ATT TCA GGT GCC CAY TYV GGN GT |  |  |  |  |  |  |
| *arsM* | ArsMF1 | TCY CTC GGC TGC GGC AAY CCV AC | 0.3 | 60 | 346 | | *Terriglobus roseus* | Jia et al., 2013 |
|  | ArsMF2 | CGW CCG CCW GGC TTW AGY ACC CG |  |  |  |  |  |  |
| *dsrA* | Dsr1+-F | ACS CAC TGG AAG CAC GGC GG | 0.3 | 59 | 221 | | *Desulfobacterium autotrophicum* | Merlino et al., 2013 |
|  | Dsr-R | GTG GMR CCG TGC AKR TTG G |  |  |  |  |  |  |
| *Geobacteraceae* | Geo564F | AAG CGT TGT TCG GAW TTA T | 0.3 | 60 | 276 | | Clone Geo6 | Cummings et al., 2003 |
|  | Geo840R | GGC ACT GCA GGG TCA ATA |  |  |  |  |  |  |
| *Shewanellaceae* | She120F | GCC TAG GGA TCT GCC CAG TCG | 0.3 | 60 | 100 | Clone Shew1 | | Himmelheber, 2009 |
|  | She220R | CTA GGT TCA TCC AAT CGC G |  |  |  |  |  |  |
| *Gallionellaceae* | 628F | GBM AGG CTA GAG TGT AGC | 0.3 | 56 | 370 | Clone GAL1 | | Wang et al., 2009 |
|  | 998R | CTC TGG AAA CTT CCT GAC |  |  |  |  |  |  |

**Supplementary Table 6** Results of processing of Illumina 16S rRNA genes sequences. F: Fornazzo soil; V: Veronica soil B: *Bacteria*; A: *Archaea*; C: control without sulfate; S: sulfate amendment; RS: rhizosphere soil; PW: pore water; T0: original unplanted soil (before seeding); T1: stem elongation; T2: flowering; T3: dough.

| **Sample** | **Raw reads** | **Filtered reads** | **Number of ASVs** |
| --- | --- | --- | --- |
| F-B-T0 | 50095 ± 8440 | 26477 ± 5792 | 117.33 ± 12.42 |
| F-B-C-RS-T1 | 35168 ± 1783 | 12831 ± 1384 | 118.00 ± 6.00 |
| F-B-S-RS-T1 | 35643 ± 1012 | 13356 ± 776 | 115.67 ± 10.60 |
| F-B-C-RS-T2 | 35914 ± 2562 | 11965 ± 2762 | 111.00 ± 11.53 |
| F-B-S-RS-T2 | 36302 ± 1298 | 13019 ± 1318 | 116.67 ± 5.69 |
| F-B-C-RS-T3 | 35099 ± 1008 | 13621 ± 350 | 120.67 ± 3.51 |
| F-B-S-RS-T3 | 35943 ± 313 | 13230 ± 922 | 119.33 ± 7.23 |
| F-B-C-PW-T2 | 32505 ± 2489 | 12858 ± 1525 | 75.00 ± 1.41 |
| F-B-S-PW-T2 | 34848 ± 2432 | 13700 ± 1667 | 83.00 ± 8.49 |
| F-B-C-PW-T3 | 29963 ± 2503 | 10433 ± 2846 | 66.00 ± 1.41 |
| F-B-S-PW-T3 | 33469 ± 146 | 12578 ± 936 | 63.50 ± 2.12 |
| V-B-T0 | 54121 ± 5343 | 25775 ± 2292 | 107.33 ± 4.16 |
| V-B-C-RS-T1 | 38777 ± 5095 | 14801 ± 2581 | 113.00 ± 7.21 |
| V-B-S-RS-T1 | 35563 ± 2760 | 13013 ± 978 | 108.00 ± 1.00 |
| V-B-C-RS-T2 | 36020 ± 2361 | 14314 ± 921 | 113.67 ± 7.09 |
| V-B-S-RS-T2 | 36408 ± 2525 | 14401 ± 1731 | 113.33 ± 11.15 |
| V-B-C-RS-T3 | 34685 ± 768 | 12572 ± 530 | 102.00 ± 6.24 |
| V-B-S-RS-T3 | 34976 ±1721 | 13296 ± 1182 | 106.00 ± 8.66 |
| V-B-C-PW-T2 | 34807 | 15853 | 88.00 |
| V-B-S-PW-T2 | 35994 ± 5195 | 18821 ± 2700 | 92.50 ± 6.36 |
| V-B-C-PW-T3 | 36311 ± 4270 | 15684 ± 2838 | 85.50 ± 4.95 |
| V-B-S-PW-T3 | 36263 ± 3846 | 17611 ± 3085 | 95.00 ± 8.49 |
| F-A-T0 | 67379 ± 2662 | 22546 ± 1591 | 35.67 ± 2.08 |
| F-A-C-RS-T1 | 66821 ±3889 | 24568 ± 3025 | 56.67 ± 3.21 |
| F-A-S-RS-T1 | 50693 ± 33407 | 17755 ± 12547 | 47.67 ± 13.05 |
| F-A-C-RS-T2 | 69754 ± 2307 | 22246 ± 2334 | 50.00 ± 3.46 |
| F-A-S-RS-T2 | 67423 ± 4143 | 22452 ± 643 | 49.33 ± 1.53 |
| F-A-C-RS-T3 | 68244 ± 602 | 23424 ± 815 | 52.33 ± 6.43 |
| F-A-S-RS-T3 | 69570 ± 2653 | 22688 ± 1766 | 49.33 ± 5.13 |
| F-A-C-PW-T2 | 64592 ± 2665 | 28441 ± 2253 | 43.50 ± 2.12 |
| F-A-S-PW-T2 | 64771 ± 4332 | 25625 ± 2490 | 46.50 ± 3.54 |
| F-A-C-PW-T3 | 70661 ± 1984 | 32451 ± 1978 | 49.50 ± 4.95 |
| F-A-S-PW-T3 | 63600 ± 1668 | 27450 ± 19 | 47.50 ± 0.71 |
| V-A-T0 | 65464 ± 6823 | 25638 ± 3042 | 48.67 ± 1.53 |
| V-A-C-RS-T1 | 71231 ± 5634 | 24442 ± 2166 | 56.67 ± 5.51 |
| V-A-S-RS-T1 | 68825 ± 4068 | 21752 ± 1644 | 52.33 ± 1.53 |
| V-A-C-RS-T2 | 69086 ± 3010 | 22612 ± 1789 | 48.67 ± 3.51 |
| V-A-S-RS-T2 | 68880 ± 956 | 21917 ± 1187 | 47.67 ± 2.52 |
| V-A-C-RS-T3 | 69726 ± 3973 | 20856 ± 3306 | 50.33 ± 2.08 |
| V-A-S-RS-T3 | 66028 ± 1811 | 21564 ± 1188 | 49.33 ± 3.79 |
| V-A-C-PW-T2 | 73837 | 33195 | 43.00 |
| V-A-S-PW-T2 | 61111 ± 3587 | 29037 ± 1129 | 39.00 ± 4.24 |
| V-A-C-PW-T3 | 71244 ± 6947 | 31191 ± 3763 | 46.50 ± 4.95 |
| V-A-S-PW-T3 | 63753 ± 506 | 29212 ± 1333 | 49.00 ± 4.24 |

**Supplementary Table 7** Results of the Mantel test applied to the Redundancy Analysis (RDA) performed between the beta diversity of bacterial and archaeal communities in rhizosphere soil and pore water and the related physicochemical parameters listed in Supplementary Tables 2 and 4 and the target genes quantified with RT-qPCR.

|  | **Bacteria** | | **Archaea** | |
| --- | --- | --- | --- | --- |
| **Factor** | **Rhizosphere soil** | **Pore water** | **Rhizosphere soil** | **Pore water** |
| TIC | 0.84^***^ | 0.44^**^ | 0.53^**^ | 0.73^**^ |
| TOC | 0.36^**^ | -0.18 | 0.33^*^ | 0.01 |
| Total sulfur | 0.70^**^ | 0.51^**^ | 0.45^**^ | 0.58^*^ |
| Ferrous iron | 0.63^**^ | 0.62^**^ | 0.55^**^ | 0.58^*^ |
| Eh | 0.09 | -0.35 | -0.02 | 0.08 |
| pH | 0.35^*^ | 0.48^*^ | 0.15 | 0.42^*^ |
| Total arsenic | 0.22^*^ | 0.02 | 0.18 | 0.35 |
| Methylated arsenic | -0.04 | -0.01 | -0.15 | 0.05 |
| Methylated oxyarsenates | -0.06 | -0.05 | -0.14 | 0.06 |
| Total thioarsenates | 0.06 | -0.28 | -0.03 | 0.10 |
| Inorganic thioarsenates | -0.01 | -0.27 | 0.09 | -0.09 |
| Methylated thioarsenates | 0.11 | 0.19 | -0.04 | 0.43^*^ |
| *arsC* | -0.03 | 0.13 | -0.03 | 0.13 |
| *aioA* | -0.08 | - | -0.08 | - |
| *arsM* | 0.41^**^ | 0.45^*^ | 0.41^*^ | 0.45^*^ |
| *Geobacteraceae* | 0.02 | 0.16 | 0.02 | 0.16 |
| *Shewanellaceae* | 0.26^*^ | 0.61^**^ | 0.26^*^ | 0.61^**^ |
| *Gallionellaceae* | 0.11 | 0.52^*^ | 0.11 | 0.52^*^ |
| *dsr* | 0.04 | - | 0.04 | - |

^*^ p ≤ 0.05; ^**^ p ≤ 0.01; ^***^ p ≤ 0.001

**Supplementary Table 8** Results of the Mantel test applied to the Redundancy Analysis (RDA) performed between the distribution in the samples of microbial populations involved in arsenic, iron and sulfur cycles according to dataset 1 in rhizosphere soil and pore water and the related physicochemical parameters listed in Supplementary Tables 2 and 4 and the target genes quantified with RT-qPCR.

| **Factor** | **Rhizosphere soil** | **Pore water** |
| --- | --- | --- |
| TIC | 0.60^**^ | 0.07 |
| TOC | 0.57^**^ | -0.26 |
| Total sulfur | 0.60^***^ | 0.29^*^ |
| Ferrous iron | 0.50^**^ | 0.15 |
| Eh | -0.05 | -0.30 |
| pH | 0.25^*^ | 0.08 |
| Total arsenic | 0.01 | -0.31 |
| Methylated arsenic | 0.12 | -0.15 |
| Methylated oxyarsenates | 0.12 | -0.12 |
| Total thioarsenates | -0.03 | -0.14 |
| Inorganic thioarsenates | -0.01 | -0.01 |
| Methylated thioarsenates | 0.17 | -0.37 |
| *arsC* | 0.04 | 0.60^**^ |
| *aioA* | -0.14 | - |
| *arsM* | 0.63^***^ | 0.57^**^ |
| *Geobacteraceae* | 0.17 | -0.11 |
| *Shewanellaceae* | 0.01 | -0.03 |
| *Gallionellaceae* | -0.06 | 0.44 |
| *dsr* | 0.07 | - |

^*^ p ≤ 0.05; ^**^ p ≤ 0.01; ^***^ p ≤ 0.001

**Supplementary Table 9** Results of the Mantel test applied to the Redundancy Analysis (RDA) performed between of the distribution of the functionalities involved in arsenic iron and sulfur cycles according to Tax4Fun2 analysis in rhizosphere soil and pore water and the related physicochemical parameters listed in Supplementary Tables 2 and 4 and the target genes quantified with RT-qPCR.

| **Factor** | **Rhizosphere soil** | **Pore water** |
| --- | --- | --- |
| TIC | 0.39^**^ | 0.24 |
| TOC | 0.61^***^ | -0.04 |
| Total sulfur | 0.26^*^ | 0.33^*^ |
| Ferrous iron | 0.28^*^ | 0.31^*^ |
| Eh | 0.20 | 0.02 |
| pH | 0.25 | -0.06 |
| Total arsenic | -0.08 | 0.09 |
| Methylated arsenic | 0.21 | 0.45^*^ |
| Methylated oxyarsenates | 0.15 | 0.49^*^ |
| Total thioarsenates | 0.03 | -0.26 |
| Inorganic thioarsenates | 0.05 | -0.23 |
| Methylated thioarsenates | 0.26^*^ | -0.25 |
| *arsC* | 0.01 | 0.34^*^ |
| *aioA* | 0.13 | - |
| *arsM* | 0.47^**^ | 0.35 |
| *Geobacteraceae* | 0.11 | -0.23 |
| *Shewanellaceae* | 0.11 | 0.13 |
| *Gallionellaceae* | 0.03 | 0.07 |
| *dsr* | 0.19 | - |

^*^ p ≤ 0.05; ^**^ p ≤ 0.01; ^***^ p ≤ 0.001

**Supplementary Table 10** Pearson correlation among microbial populations involved in arsenic, iron and sulfur cycles and physico-chemical parameters.

|  | **Rhizosphere soil** | | | | | **Pore water** | | | | | |
| --- | --- | --- | --- | --- | --- | --- | --- | --- | --- | --- | --- |
| **Parameter** | **DAsRB** | **FeOB** | **AsOB** | **DSRB** | **SOB** | | **DAsRB** | **FeOB** | **AsOB** | **DSRB** | **SOB** |
| As | -1.79 | 0.06 | -1.95 | -1.09 | -0.78 | | 0.04 | -1.20 | -0.32 | 0.69 | -0.65 |
| Fe(II) | -1.91 | 1.76 | -2.75^*^ | -4.28^**^ | -0.76 | | 0.48 | -3.46^*^ | -0.66 | -1.14 | -0.09 |
| Tot S | -3.05^*^ | 2.85^*^ | -3.71^**^ | -4.34^**^ | 0.34 | | 1.05 | -2.56^*^ | -0.01 | -1.06 | -0.70 |
| pH | -2.19 | 2.10 | -2.14 | -1.75 | 1.06 | | 0.36 | -1.59 | -0.42 | -1.53 | -0.45 |
| Eh | 1.13 | -1.39 | 0.95 | 0.02 | -2.19 | | -0.01 | -0.27 | -0.21 | -0.13 | 0.16 |
| TIC | -3.07^*^ | 2.39^*^ | -3.95^**^ | -3.37^**^ | 0.28 | | 0.63 | -2.12 | -0.22 | -1.01 | -0.53 |
| TOC | -4.13^**^ | 2.34^*^ | -4.47^**^ | -2.84^*^ | 0.34 | | -0.26 | -0.49 | -0.24 | -0.21 | -0.96 |
| Meth As  (%) | 0.91 | -0.42 | 1.39 | 2.03 | 2.08 | | -0.31 | 2.58^*^ | 0.70 | -0.24 | -0.91 |
| Meth As(III) (%) | 1.11 | -0.55 | 1.60 | 2.08 | 1.82 | | -0.41 | 2.36 | 0.58 | -0.10 | -0.92 |
| Tot ThioAs (%) | 0.13 | 0.90 | 0.60 | 1.38 | 3.59^**^ | | 0.04 | 0.94 | 0.40 | -0.66 | 0.31 |
| Inorg ThioAs (%) | 0.41 | 1.00 | 0.84 | 1.15 | 2.37^*^ | | -0.27 | -0.26 | -0.48 | -0.07 | 1.15 |
| Meth ThioAs (%) | -0.32 | 0.41 | 0.05 | 1.11 | 2.95^*^ | | 0.32 | 2.13 | 1.16 | -0.99 | -0.54 |
| Meth As (ppb) | -0.64 | 0.12 | -0.37 | 0.33 | 1.23 | | -0.51 | 1.71 | 0.41 | 0.01 | -1.33 |
| Meth As(III) (ppb) | -0.39 | -0.14 | -0.16 | 0.40 | 0.94 | | -0.61 | 1.65 | 0.31 | 0.21 | -1.38 |
| Tot ThioAs (ppb) | -1.66 | 2.59^*^ | -1.19 | -0.16 | 3.17^**^ | | -0.01 | -0.12 | -0.03 | -0.27 | 0.26 |
| Inorg ThioAs (ppb) | -1.08 | 2.17 | -0.71 | -0.09 | 2.25^*^ | | -0.16 | -0.80 | -0.51 | 0.29 | 0.65 |
| Meth ThioAs (ppb) | -1.70 | 1.53 | -1.38 | -0.20 | 2.21 | | 0.21 | 0.96 | 0.71 | -1.08 | -0.41 |

^*^ p ≤ 0.05; ^**^ p ≤ 0.01

**2. Supplementary figures**


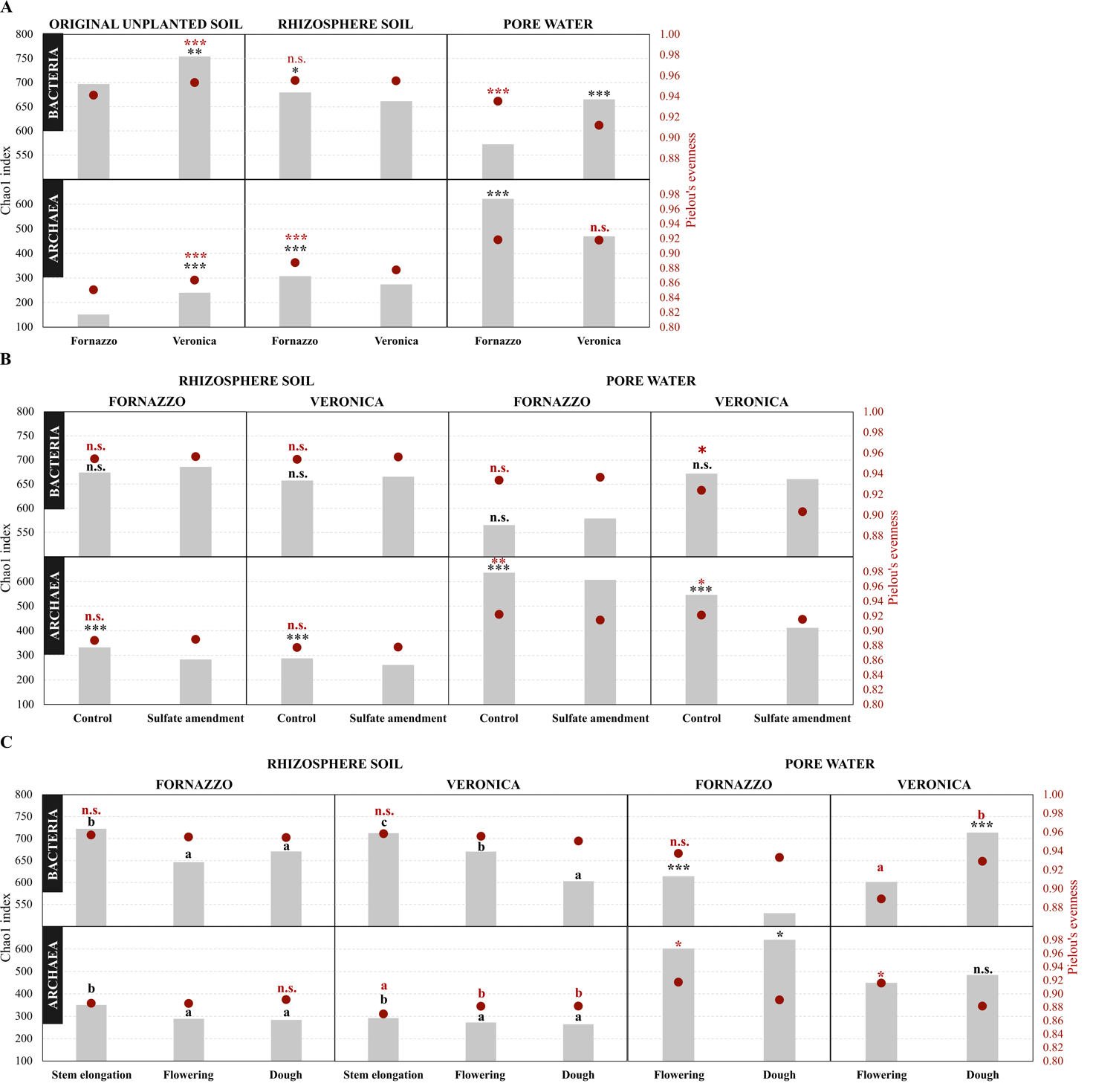


**Supplementary Figure 1** Alpha diversity by means of Chao1 index and Pielou’s evenness of bacterial and archaeal communities in the original unplanted soil, rhizosphere soil and pore water from Veronica soil with and without sulfate and from Fornazzo soil with and without sulfate. Comparisons were statistically tested to evaluate the soil type (A), sulfate amendment (B) and time (C) effects (letters indicate significantly different groups according to ANOVA; asterisks indicate statistical significance according to t test; * p ≤ 0.05; ** p ≤ 0.01; *** p ≤ 0.001).


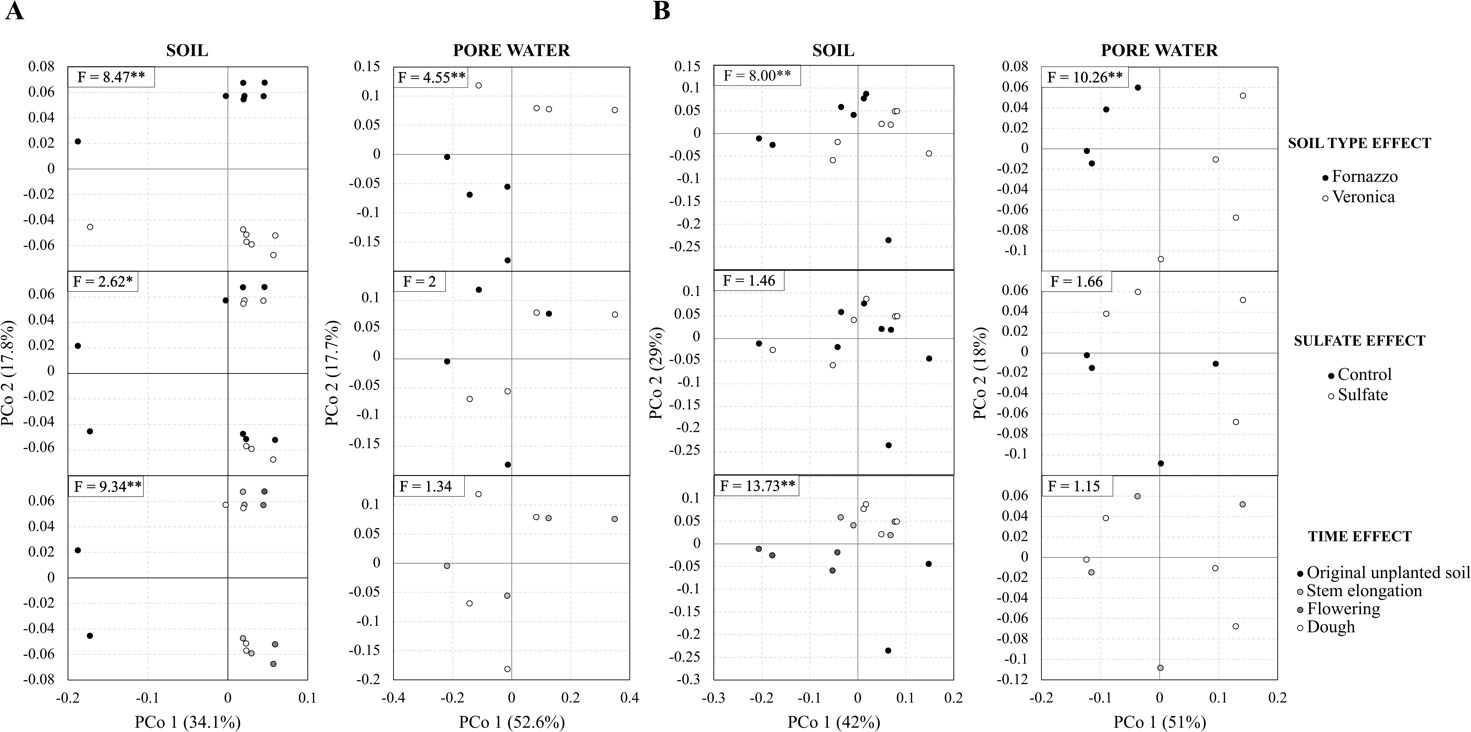


**Supplementary Figure 2** Beta diversity by means of weighted UniFrac dissimilarities of bacterial (A) and archaeal (B) communities in the original unplanted soil, rhizosphere soil and pore water from Veronica soil with and without sulfate and from Fornazzo soil with and without sulfate, at stem elongation, flowering, and dough. The PERMANOVA test (F) indicates the statistical significance of sample groups defined by the compartment, soil type and sulfate amendment (* p ≤ 0.05; ** p ≤ 0.01).


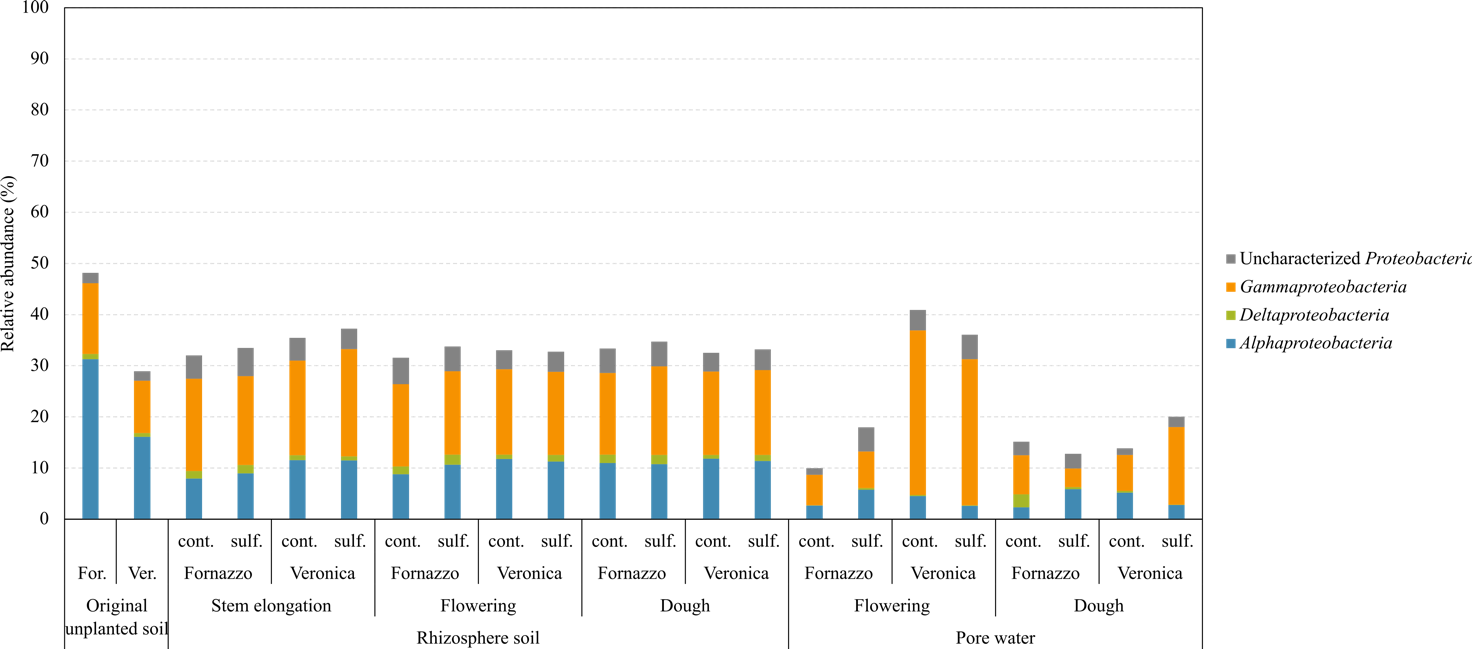


**Supplementary Figure 3** Relative abundance of the main classes within the phylum *Proteobacteria* retrieved with Illumina 16S rRNA gene sequencing.


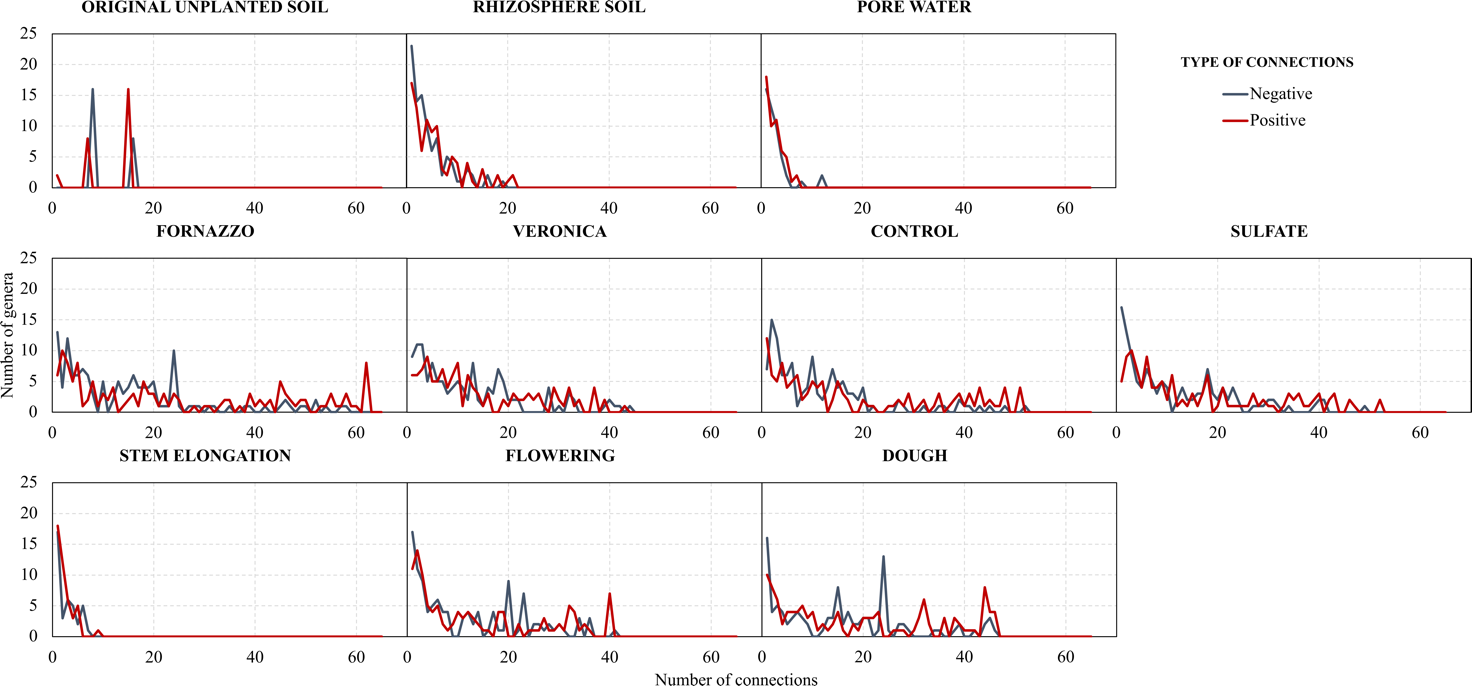


**Supplementary Figure 4** Number of genera that showed positive and negative connections with other genera in each group.


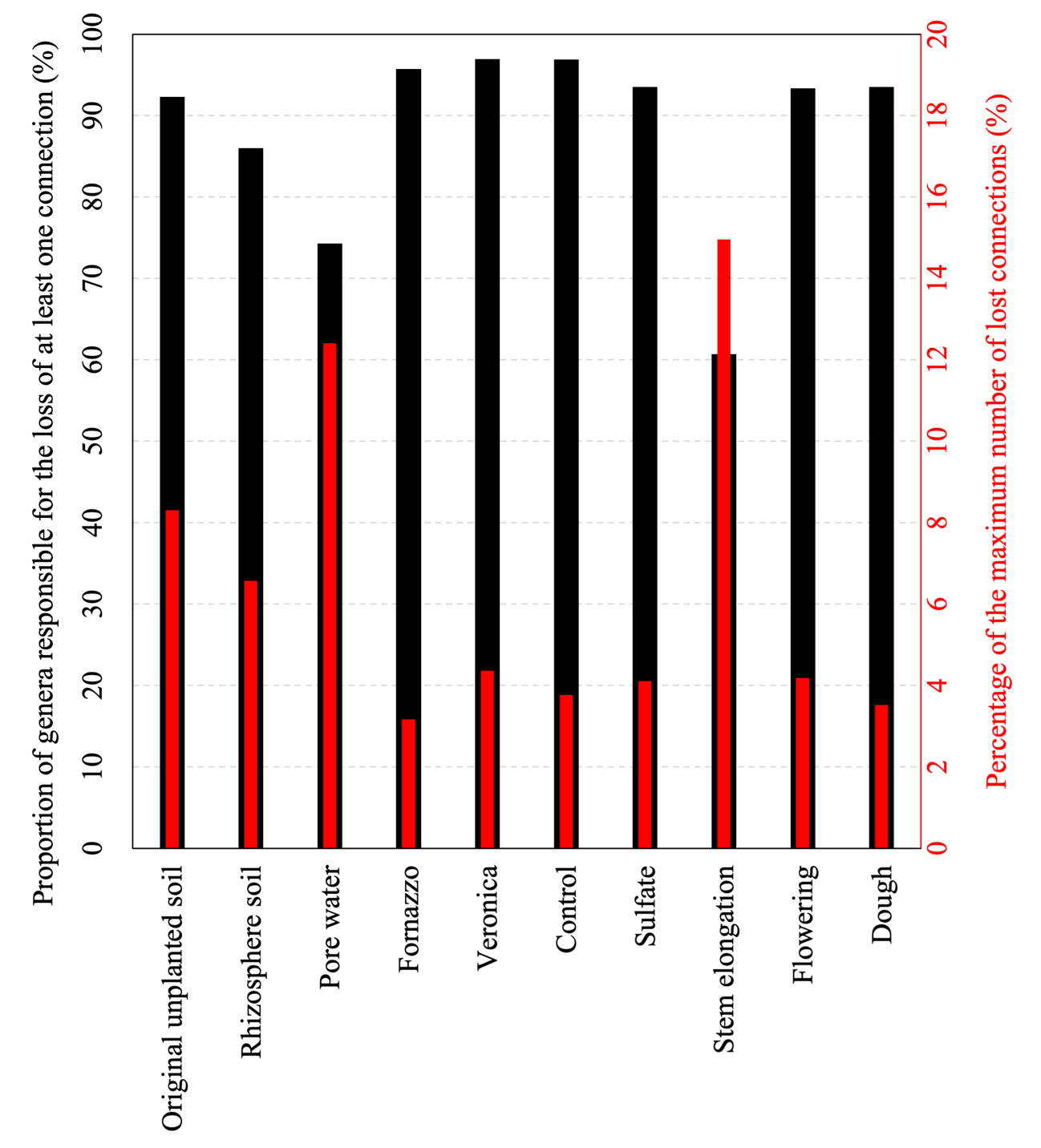


**Supplementary Figure 5** For each network obtained with co-occurrence analysis, the proportion of genera responsible for the loss of at least one connection (black bars) and the percentage of the maximum number of lost connection (red bars) was calculated.


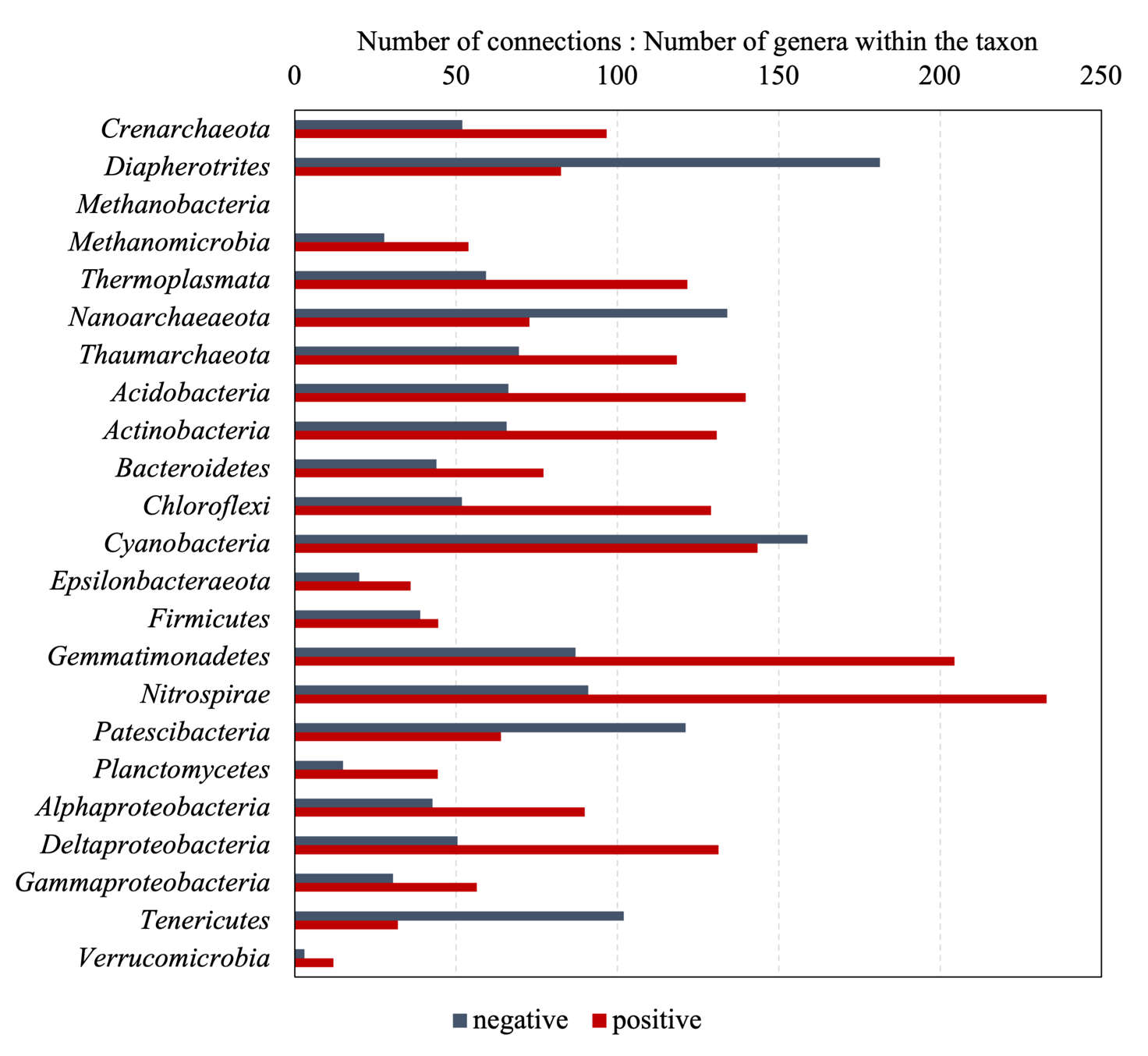


**Supplementary Figure 6** Ratio between the total number of correlations and the number of connected genera within each taxon (i.e., class/phylum).


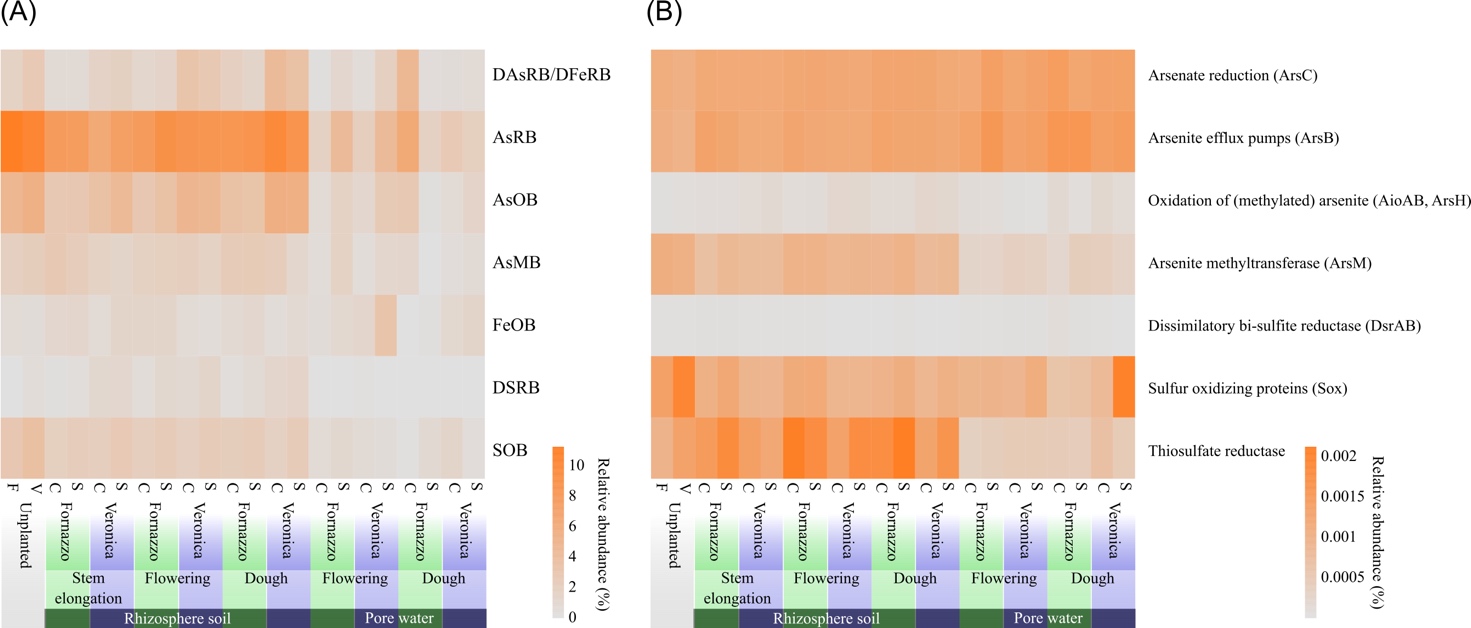


**Supplementary Figure 7** Relative abundance of genera including dissimilatory arsenate-reducing bacteria (DAsRB), arsenate-reducing bacteria (AsRB), arsenite-oxidizing bacteria (AsOB), arsenite-methylating bacteria (AsMB), dissimilatory sulfate-reducing bacteria (DSRB), sulfur-oxidizing bacteria (SOB), dissimilatory Fe(III)-reducing bacteria (DFeRB) and Fe(II)-oxidizing bacteria (FeOB) (A) and relative abundance of enzymes involved in detoxifying arsenate-reduction (i.e., ArsC), arsenite extrusion from the cell (i.e., ArsB), arsenite oxidation (i.e., AioAB) and arsenite methylation (i.e., ArsM and ArsH) (B). F, Fornazzo soil, V; Veronica soil; C, control; S, sulfate amendment.

**3. References**

Bachate, S.P., Cavalca, L., Andreoni, V., 2009. Arsenic-resistant bacteria isolated from agricultural soils of Bangladesh and characterization of arsenate-reducing strains. Journal of Applied Microbiology 107, 145–156. https://doi.org/10.1111/j.1365-2672. 2009.04188.x

Cummings, D.E., Snoeyenbos-West, O.L., Newby, D.T., Niggemyer, A.M., Lovley, D.R., Achenbach, L.A., et al., 2003. Diversity of Geobacteraceae species inhabiting metal-polluted freshwater lake sediments ascertained by 16S rDNA analyses. Microbial Ecology 46(2), 257-269. https://doi.org/10.1007/s00248-002-0005-8

Fierer, N., Jackson, J.A., Vilgalys, R., Jackson, R.B., 2005. Assessment of soil microbial community structure by use of taxon-specific quantitative PCR assays. Applied and Environmental Microbiology 71(7), 4117-4120. https://doi.org/10.1128/AEM.71.7.4117-4120.2005

Himmelheber, D.W., Thomas, S.H., Löffler, F.E., Taillefert, M., Hughes, J.B., 2009. Microbial colonization of an *in situ* sediment cap and correlation to stratified redox zones. Environmental Science & Technology 43(1), 66-74. https://doi.org/10.1021/es801834e

Jia, Y., Huang, H., Zhong, M., Wang, F.H., Zhang, L.M., Zhu, Y.G., 2013. Microbial arsenic methylation in soil and rice rhizosphere. Environmental Science & Technology 47(7), 3141-3148. https://doi.org/10.1021/es303649v

Malasarn, D., Saltikov, C.W., Campbell, K.M., Santini, J.M., Hering, J.G., Newman, D.K., 2004. *arrA* is a reliable marker for As(V) respiration. Science, 306(5695), 455-455. https://doi.org/10.1126/science.110237

Merlino, G., Rizzi, A., Schievano, A., Tenca, A., Scaglia, B., Oberti, R., et al., 2013. Microbial community structure and dynamics in two-stage vs single-stage thermophilic anaerobic digestion of mixed swine slurry and market bio-waste. Water Research 47(6), 1983-1995. https://doi.org/10.1016/j.watres.2013.01.007

Quéméneur, M., Cébron, A., Billard, P., Battaglia-Brunet, F., Garrido, F., Leyval, C., et al., 2010. Population structure and abundance of arsenite-oxidizing bacteria along an arsenic pollution gradient in waters of the upper isle River Basin, France. Applied and Environmental Microbiology 76(13). 4566-4570. https://doi.org/10.1128/AEM.03104-09

Quéméneur, M., Heinrich-Salmeron, A., Muller, D., Lièvremont, D., Jauzein, M., Bertin, P.N., et al., 2008. Diversity surveys and evolutionary relationships of *aoxB* genes in aerobic arsenite-oxidizing bacteria. Applied and Environmental Microbiology 74(14), 4567-4573. https://doi.org/10.1128/AEM.02851-07

Wang, J., Kerl, C. F., Hu, P., Martin, M., Mu, T., Brüggenwirth, L., Wu, G., Said-Pullicino, D., Romani, M., Wu, L., Planer-Friedrich, B., 2020. Thiolated arsenic species observed in rice paddy pore waters. Nature Geoscience, 13(4), 282-287. https://doi.org/10.1038/s41561-020-0533-1

Wang, J., Muyzer, G., Bodelier, P.L.E., Laanbroek, H.J., 2009. Diversity of iron oxidizers in wetland soils revealed by novel 16S rRNA primers targeting *Gallionella*-related bacteria. ISME Journal 3, 715-725. https://doi.org/10.1038/ismej.2009.7

Yu, Y., Lee, C., Kim, J., Hwang, S., 2005. Group‐specific primer and probe sets to detect methanogenic communities using quantitative real‐time polymerase chain reaction. Biotechnology and Bioengineering 89(6), 670-679. https://doi.org/10.1002/bit.20347
